# Caprylate Salts Based on Amines as Volatile Corrosion Inhibitors for Metallic Zinc: Theoretical and Experimental Studies

**DOI:** 10.3389/fchem.2017.00032

**Published:** 2017-05-31

**Authors:** Marco A. G. Valente, Deiver A. Teixeira, David L. Azevedo, Gustavo T. Feliciano, Assis V. Benedetti, Cecílio S. Fugivara

**Affiliations:** ^1^Departamento de Físico-Química, Instituto de Química, Universidade Estadual Paulista (Unesp)Araraquara, Brazil; ^2^Departamento de Campus Cuiabá (IFMT), Instituto Federal do Mato GrossoCuiabá, Brazil; ^3^Instituto de Física, Universidade de BrasíliaBrasília, Brazil

**Keywords:** zinc, EIS, modeling studies, neutral medium inhibitor

## Abstract

The interaction of volatile corrosion inhibitors (VCI), caprylate salt derivatives from amines, with zinc metallic surfaces is assessed by density functional theory (DFT) computer simulations, electrochemical impedance (EIS) measurements and humid chamber tests. The results obtained by the different methods were compared, and linear correlations were obtained between theoretical and experimental data. The correlations between experimental and theoretical results showed that the molecular size is the determining factor in the inhibition efficiency. The models used and experimental results indicated that dicyclohexylamine caprylate is the most efficient inhibitor.

## Introduction

It is well-known that many metal parts are produced far from where they are used and/or need to be stored for a long time. In other cases, the metals pieces such as automobile underbodies, offshore drilling decks, storage tanks, naval vessels, and those used in the petrochemical industry are subject to aggressive conditions. Under these conditions they are subjected to corrosion due to the aggressiveness of the environment. Therefore, these materials need to be protected against corrosion and this protection is generally performed by volatile corrosion inhibitors (VCI), that are often impregnated in plastic films involving the metal pieces (Vuorinen et al., 2004). However, many of these compounds like amines cannot be incorporated in plastic or sachets and, therefore, their salts are used. It is also noted that sodium salts have too small vapor pressure to be used as VCI and some amines (cyclohexylamine, ethanolamine) have so high vapor pressure that can only protect the material for very short time. If only amines constitute the vapor, the pH at the metallic surface should be to high (pH > 10) to protect metals like Zn, Mg, and Al because they are corroded in this pH region, which is another reason to use these amines in the ammonium salt form. Ammonium salts of carboxylic acids are also sublimable and some salts of weak carboxylic acids act on defected sites in the natural oxide layer suppressing the corrosion process (Rammelt et al., 2009).

The action of the VCI on the metal surface can occur in two main ways (Bastidas et al., 2005a): (a) dissociation of the VCI before reaching the metal surface and saturation of the atmosphere in contact with the material to be protected; (b) the VCI molecules are volatilized without dissociating, and they are only dissociated when attaining the metal surface. The vapor of VCI may saturate the air surrounding the metal or inside the container and adsorb on the metal surface to form a protective film. It is known that, in the presence of humidity, a thin aqueous film may be formed on the metal surface. The VCI dissolved in this film can provide protection against corrosion always when the pH of this solution is approximately neutral, i.e., neither excessively acidic nor alkaline. Therefore, only VCI of weak acids and salts derived from volatile bases, such as amines, would be the best candidates to be used as VCIs.

Many organic molecules containing an amino functional group have been investigated and used as corrosion inhibitors for the zinc surface (Subramanian et al., 1999; Rammelt et al., 2009; Teixeira et al., 2015). The high electron density and the presence of oxygen, sulfur, nitrogen, and π-bonds are the main characteristics of these molecules tested as corrosion inhibitors (Quraishi et al., 2002; Vuorinen et al., 2004). Besides, they must have adequate vapor pressure to fill the environment and keep high efficiency against corrosion, which means maintain the metal parts completely covered with the corrosion inhibitor for the time required (Bastidas et al., 2005a).

It is well-known that the quantity of amines that suppress completely the corrosion of zinc is less than that suppress the corrosion of steel due to the nature of the interaction between the metal and the amine. In steels the iron has incomplete sublevels while zinc has completed sublevels, then, the adsorption may be only result of electron exchange. In the presence of chloride ions into the solution, in general, the inhibitor capability of the amines to prevent zinc corrosion increases with the increase in basicity or dipole moment of the amine (Subramanian et al., 2000).

The vapor pressure of VCI ranges from 10 to 10^−4^ Pa at atmospheric pressure (Andreev and Ibatullin, 2002). Different experimental methods have been used to determine the vapor pressure of a VCI and its dependence on temperature. It is known that the vapor pressure values available show certain inconsistencies and the reproducibility depends on the experimental parameters. These parameters are intrinsic characteristics of the method used (Bastidas et al., 2005a). These inconsistencies in vapor pressure values limit a better understanding of the VCI mechanism.

The efficiency of VCI tests can be investigated, among others, using the wet chamber (Skinner, 1993; Wan et al., 2005), electrochemical tests (Rammelt et al., 2009, 2011), and theoretical and computational treatment (Teixeira et al., 2015).

Humid chamber experiments reproduce environmental conditions of humidity and temperature cycles, requiring many days for testing. Electrochemical techniques, such as electrochemical impedance spectroscopy (EIS) are relatively fast, and provide information about the relevant chemical processes that might occur on the surface. Computer simulations often offer mechanistic interpretations about the observed phenomena, when combined with experimental techniques. When the fundamental interactions among the atomic constituents are known, properties such as interaction energy, charge distribution, and electronegativity can be evaluated from model systems, and thus, a picture for the process of metal surface protection against corrosion is obtained (Gece, 2008; Bürger et al., 2013; Jafari et al., 2013). Furthermore, other works suggest that there is a correlation between the protective properties of the VCIs with their electronic structure (Bentiss et al., 2003; Gece, 2008; Outirite et al., 2010), in particular, the energetic alignment of the frontier molecular orbitals and the electric dipole moment.

In order to reduce even more the computational cost of the calculations, the ONIOM (Our own N-layered Integrated Molecular Orbital and mechanics Molecular) model is used, throughout the work (Dapprich et al., 1999) where the system can be partitioned into layers and the part of the system of most interest, i.e., where the reaction actually takes place, can be treated at a high level of theory. Consequently, the other layers of the system may be treated at different levels, for example, classic level, semi-empirical, or quantum even with different base functions (Rassolov et al., 1998).

The VCI is of great significance for the metallurgical industry and the development of more efficient inhibitors for different materials and environmental conditions represents a challenge for scientific and engineering researchers. In the study of new VCIs, an important point to be considered is the metallic surface, which is normally not included in the theoretical calculations due to the difficulty to stablish the main parameters, the time consume and cost.

The present work aims to theoretically correlate the structures and chemical properties of inhibitors based on ammonium caprylate salts of cyclohexylamine (CCHA), dicyclohexylamine (CDCHA), and ethanolamine (CETA) with the metallic surface, and compare with the experimental results. For that the interactions between zinc surface and VCIs were investigated in humidity chamber, and by means of electrochemical impedance spectroscopy (EIS) measurements in the absence and presence of VCI. The quantitative structure-activity relationship (QSAR) was obtained from the experimental electrochemical data and the theoretical data for the VCI compounds, which allowed understanding the influence of the different quantum parameters of each VCI with respect to the inhibitory activity on the metal surface (Kikuchi, 1987; Bentiss et al., 2003; Outirite et al., 2010). From information about the interactions of zinc surface with VCI molecule one can suggest the most effective VCI to act against corrosion.

## Methodology

### Theoretical studies

For computer calculations, the density functional theory (DFT) was used due to the lower computational cost when compared, for instance, to higher level calculations, and sufficient accuracy was observed in several other works (Becke, 1988; Rassolov et al., 1998; Gece, 2008). The GAUSSIAN 09W package (Frisch et al., 2009) was employed for all calculations, and the B3LYP26 (Miehlich et al., 1989) exchange correlation functional was applied, along with a 6–31G basis set (Rassolov et al., 2001). The software package GaussView (Dennington et al., 2009) was employed to determine the coordinates of the atoms, in the initial structures.

The geometry of the VCI salts, the moment of molecular dipole (μ) of frontier molecular orbitals (HOMO and LUMO), volume and footprint were obtained by adjustment of all geometric variables. The ONIOM (B3LYP/6-31g:PM6) methodology was applied to determine the interaction of each VCI with the zinc surface from the optimized unit cell size (6 × 7 × 2 Å, resulting in 157 atoms). The effect of the aqueous environment is implicitly taken into account, using the IEFPCM formalism (Cossi et al., 2003) implemented in the GAUSSIAN09W software (Frisch et al., 2009).

The periodic boundary condition is the best way to describe the metallic surface and the interaction with the VCI, disregarding undesired edge effects. However, if the cell is too small, spurious image effects are likely to occur. There is trade-off between computational cost and accuracy, specially, for geometry optimizations with analytical gradients. Moreover, the minimum region necessary for describing electronic charge transfer is the one employed in the study in its current form, and the same standard was used to all VCIs, enabling us to rank them according with the interaction strength. Other studies are under progress, but this one had, as one goal, to preliminary investigate the VCI-metal interaction.

Restrained optimization techniques are employed for obtaining potential energy surfaces (PES) as a function of the distance among the VCI molecule and the zinc surface, where a specific geometric coordinate is defined, and slowly changed, while the energy is minimized with respect to all other coordinates. In this study, the coordinate is taken to be the distance among the nitrogen atom of the VCI molecule and a reference zinc atom at the surface. For each geometry at the PES, the interaction energy *E*_int(scan VCI)_ is calculated from the energy of the entire system, *E*_total_, minus the individual energies of the VCI, *E*_VCI_, and the metal surface, *E*_surface_, at the same geometries, according to the Equation (1) (Miehlich et al., 1989; Rassolov et al., 1998):

(1) $$\begin{array}{l} E_{\text{int}\left(\text{scan VCI}\right)}\;=\;E_{\text{total}}-E_{\text{VCI}}-E_{\text{surface}} \end{array}$$

In the following step, the lateral and vertical interactions between VCIs and metal surface were analyzed, so that each system was constituted by a Zn surface extended by four times the early described, and then six molecules of VCI were added. In this case, the total interaction energy of the system, *E*_inttotal_, is a sum of the interaction energies between the VCI and the zinc surface, *E*_int(VCI/Zn surface)_ (vertical interaction), and the interaction energy between the VCI molecules, *E*_int(VCI molecules)_ (lateral interaction). Thus, the total interaction energy for each addition was obtained by Equation (2) (Rassolov et al., 2001).

(2) $$\begin{array}{l} E_{\text{int total}}\;=\;E_{\text{int}\left(\text{VCI}/\text{Zn surface}\right)}-E_{\text{int}\left(\text{VCI molecules}\right)} \end{array}$$

### Experimental studies

The cyclohexylamine (CHA), dicyclohexylamine (DCHA), and ethanolamine (ETA) compounds (Sigma Aldrich, AR grade) were used as received. The CCHA (cyclohexyl-ammonium caprylate), CDCHA (dicyclohexyl-ammonium caprylate), and CETA (ethanol-ammonium caprylate) salts were synthesized from the dissolution in ethanol of CHA, DCHA, and ETA with caprylic acid (Synth, AR) at 1:1 molar ratio, followed by drying at 50°C. Electrochemical measurements on the zinc electrode were performed in 0.1 mol L^−1^ NaCl solution (pH 6.5) with or without the addition of caprylate salts. Caprylate salts are used because amines cannot be impregnated in the sachets or plastic used to carry the VCIs as temporary protectors of materials against corrosion during their storage and/or transportation.

Zinc plates (99.9%) were polished with sandpaper of granulometry 600, 1,200, and polishing cloth with alumina slurry (particle size 0.3 μm). Afterwards, the samples were ultrasonic cleaned with isopropanol for 5 min and used as working electrodes. Thereupon, the electrochemical tests were performed in a conventional three-electrode electrochemical cell having a platinum spiral as auxiliary electrode and Ag|AgCl|KCl_sat._ as reference electrode. All electrode potentials were referred to the Ag|AgCl|KCl_sat._ electrode. The open circuit potential (E_OCP_) was recorded during 24 h. After the stabilization of the E_OCP_ of the system, the electrochemical impedance measurement was done by applying a sinusoidal signal of 10 mV (rms) to the E_OCP_ from 100 kHz to 0.050 Hz recording 10 points per frequency decade. Electrochemical measurements were conducted using a 283 EG&G PAR potentiostat and frequency response analyzer FRA 1255 Solartron controlled by an M398 PAR software. The EIS data were checked for Kramers-Kronig Transforms and analyzed with a ZView software. The reason to use aqueous solutions in this study is the fact that aqueous solutions may simulate the corrosion conditions, which are present inside the package, if a thin film of water is condensed on the metallic equipment surface.

The tests in humid chamber were performed following the standard IEC 6008-2-30 of International Electrotechnical Comission (1980). Zinc plates were subjected to wet chamber assays in the presence and absence of VCIs for 7 days. Each cycle spends 1 day: 6 h at 25°C and relative humidity (RH) of 98%, followed by a 3 h heating ramp up to 55°C; 9 h at this temperature and at RH 95%. Afterwards, the temperature was diminished to 25°C for a period of 3 h and maintained for 3 h at 25°C to close the cycle. After 7 completed cycles, the samples were visually observed and optical images obtained with an optical stereomicroscope QUIMIS.

Scanning electron microscopy images of the zinc plates surfaces were taken using a Jeol JSM 7500F SEM-FEG coupled with a Thermo Noran System six energy dispersive X ray spectrometer (EDS) before and after the immersion in 0.1 mol L^−1^ NaCl solution in the presence and absence of VCIs for 15 days.

## Results and discussion

### Theoretical results

#### Molecular orbitals

**Figure 1:**
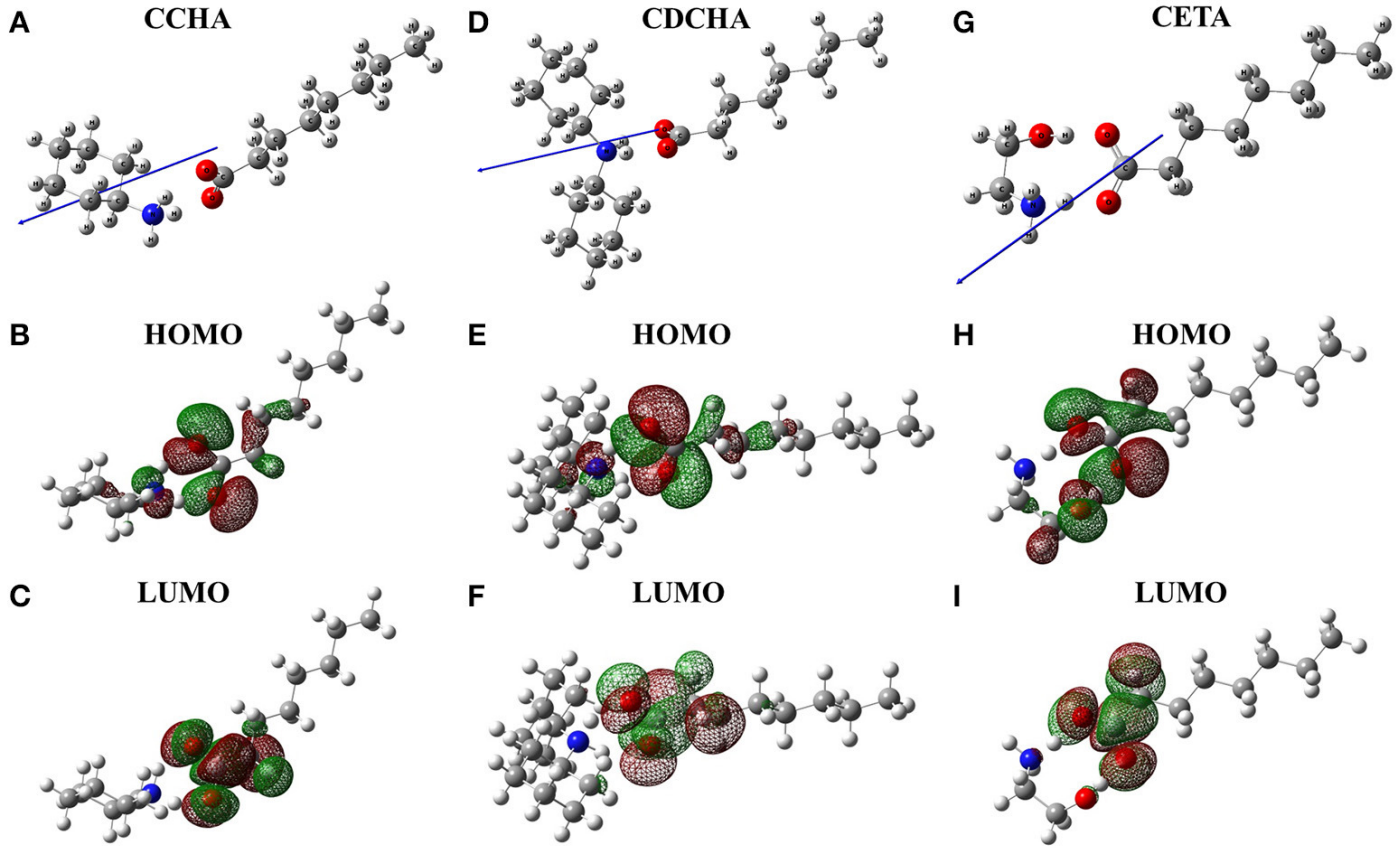
Spatial arrangement, frontier molecular orbitals and vector dipole moments (blue arrows) for caprylate salts: **(A)** CCHA-chemical structure; **(B)** CCHA-HOMO; **(C)** CCHA-LUMO; **(D)** CDCHA-chemical structure; **(E)** CDCHA-HOMO; **(F)** CDCHA-LUMO; **(G)** CETA-chemical structure; **(H)** CETA-HOMO; **(I)** CETA-LUMO.

Figure 1 shows VCIs molecules at minimum energy geometries and the location of the frontier molecular orbital (HOMO and LUMO) obtained in quantum calculations at B3LYP/6-31g level/water solvent, and in addition, the electric dipole moment vector.

The frontiers orbitals in the caprylate salts are localized on carboxylate group. In HOMO orbitals (Figures 1B,E,H) the influence of nitrogen group is noticed due to a delocalization of the wave function of this atom, except to CETA, where the wave function is localized on oxygen. However, when the LUMO orbital is observed, the electronic density is located on the anion with certain delocalization to the alkane chain (Figures 1C,F,I). Both frontier orbitals are of interest because both donor (HOMO) and acceptor (LUMO) characteristics are involved in the corrosion inhibition process (Eddy and Ebenso, 2010; Macedo et al., 2012). The vector of the dipole moment in the caprylate salts is positioned parallel to the carbonic chain of the carboxylic acid and in the direction of the nitrogen atom.

Table 1 shows the quantum-chemical parameters calculated for the interaction of VCIs with zinc. The difference between the energies of the frontier orbitals, called gaps, as well as the individual values of each orbital compared with one another, can be related to the efficiency of corrosion inhibition process. Higher energy HOMO orbitals reflect an increased tendency of donating electrons to the d orbitals of the metal, whereas lower energy LUMO orbitals will be more likely receive electrons from the d orbitals of the metal. The energy difference between HOMO and LUMO (gap) of the VCI molecules in the ground state is related to the activity of the molecule, i.e., the smaller the gap the larger the inhibition efficiency (Gece, 2008; Macedo et al., 2012). Table 1 shows that the values of the gaps obey the following order: CDCHA < CCHA < CETA.

**Table 1:** Quantum-chemical parameters calculated for the VCI interaction with zinc.

***Inhibitor***	**A_ocup_. (Å^2^/molecule)**	**μ (Debye)**	***E_HOMO_* (eV)**	***E*_LUMO_ (eV)**	**χ_inhibitor_ (eV)**	**η_inhibitor_ (eV)**	**Δ*N_Zn_***
CCHA	61.2	7.56	−6.42	1.02	2.70	3.72	0.10
CDCHA	74.2	7.81	−6.42	1.02	2.65	3.68	0.10
CETA	55.7	10.03	−6.66	0.79	2.93	3.73	0.09

The energy gap between HOMO and LUMO can be conclusive to many systems, but it must and can be better assessed when considering the interaction of these frontier orbitals with metal. For this, the Koopmans theorem (Koopmans, 1934) was used, in which the electronegativity and electron affinity can be determined and evaluated from the orbitals. In this case, the energy of the HOMO orbital is an approximation to the ionization potential (*IP*), which measures the ability of the molecule to donate its electrons. The energy of the LUMO orbital is an approximation to the electron affinity (*EA*) of the molecule, the ability to accept electrons. Thus, the electronegativity and total hardness can be obtained from *IP* (Equation 3) and *EA* (Equation 4).

(3) $$\begin{array}{l} IP=\;-E_{\text{HOMO}} \end{array}$$

(4) $$\begin{array}{l} EA=\;-E_{\text{LUMO}} \end{array}$$

Electronegativity (Equation 5) and total hardness (Equation 6).

(5) $$\begin{array}{l} \chi =\;\frac{IP+\;EA}{2} \end{array}$$

(6) $$\begin{array}{l} \eta =\;\frac{IP\;-\;EA}{2} \end{array}$$

For zinc the following reference data are found in the literature (Pearson, 1988): ionization potential *IP*_Zn_ = 9.39 eV, electron affinity *EA* = −0.49 eV, electronegativity χ_Zn_ = 4.45 eV, and total hardness η_Zn_ = 4.94 eV. Under this definition (Parr and Pearson, 1983), the chemical hardness is proportional to the second derivative of the energy with respect to the electrons in the system, and is a measure of the difficulty, or resistance to the deformation or change in the electronic structure, aligned with the concept of hard and soft acid/base theory (HSAB).

According to Equations (3–6), Table 1 shows the η and χ for VCI, and presents the fraction of electrons (Δ*N*) (Larabi et al., 2006) transferred from the inhibitor to zinc, as calculated by the (Equation 7):

(7) $$\begin{array}{l} \Delta N=\frac{\chi_{\text{Zn}}-\chi_{\text{inhibitor}}}{2\left(\eta_{\text{Zn}}+\eta_{\text{inhibitor}}\right)} \end{array}$$

Absolute hardness: companion parameter to absolute electronegativity where χ_metal_ and χ_inhibitor_ indicate the electronegativity, η_Zn_ and η_inhibitor_ the total hardness, both, respectively, to the metal and the inhibitor. Table 1 shows the quantum-chemical parameters for VCIs.

Lukovits' studies suggest a numerical criterion for the mechanism of corrosion inhibition (Wöll, 2007), if Δ*N* < 3.6 the inhibition mechanism is characterized by electronic charge transfer from the inhibitor to the metal surface, while if Δ*N* > 3.6, electron transfer takes place from the metal to the inhibitor. For inhibitors here studied and shown in Table 1, all Δ*N* values are smaller than the reference value, characterizing electronic charge transfer from the inhibitor to the metal surface.

#### Interaction of the frontier molecular orbitals of VCIs with the zinc and zinc oxide surface

**Figure 2:**
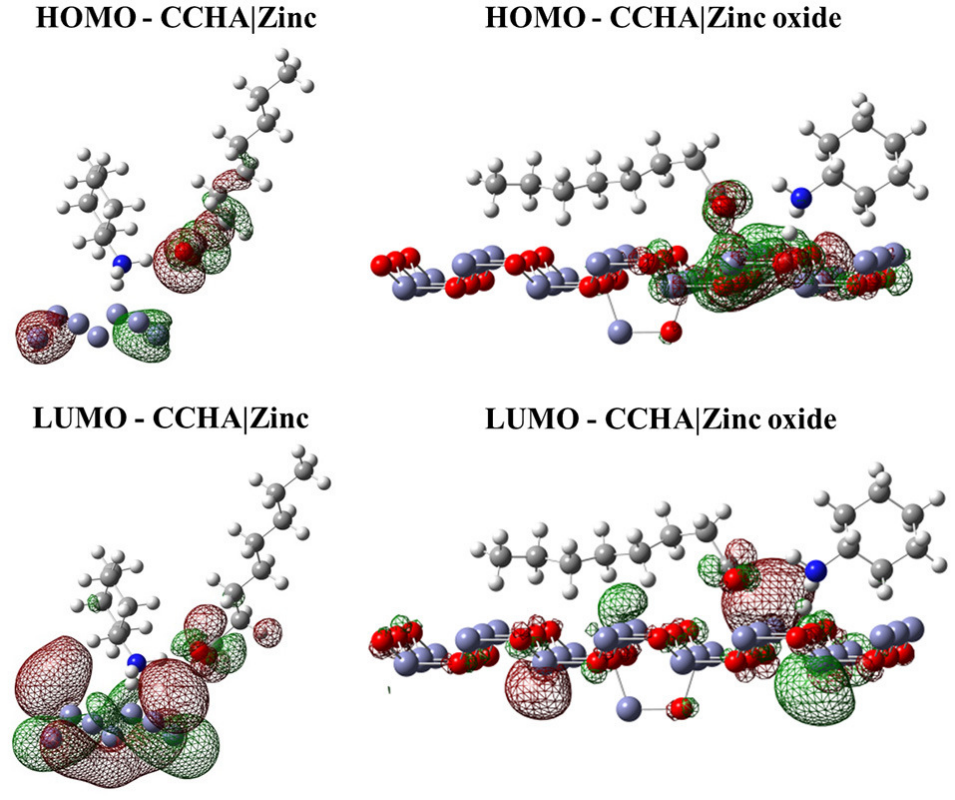
Isodensity surfaces (density value = 0.0001 A^−3^) of HOMO and LUMO orbitals of CCHA molecule with the zinc and zinc oxide surfaces. The red color represents the negative part of the wave function and the green color corresponds to the positive part.

**Figure 3:**
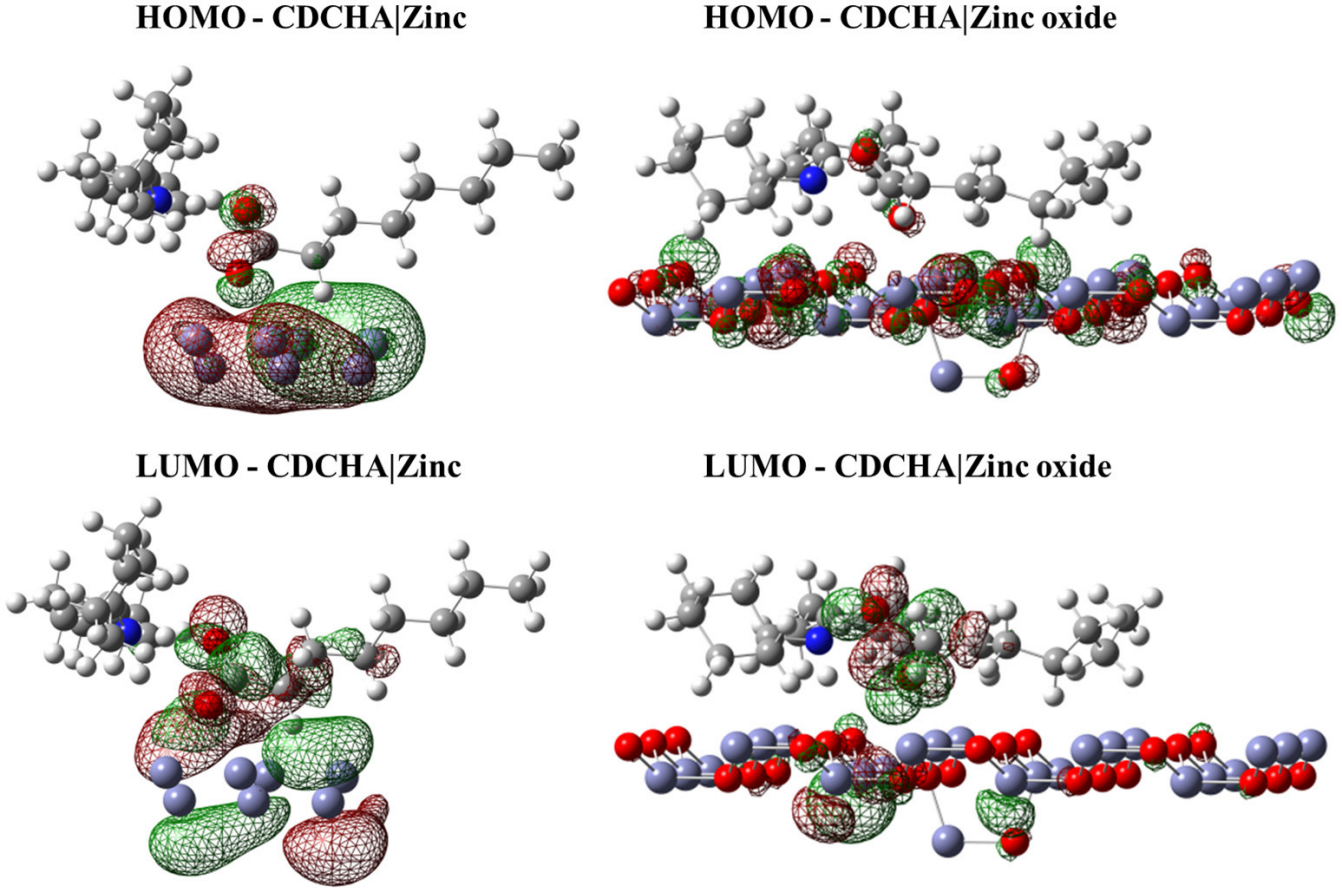
Isodensity surfaces (density value = 0.0001 A^−3^) of HOMO and LUMO orbitals of CDCHA molecule with the zinc and zinc oxide surfaces. The red color represents the negative part of the wave function and the green color corresponds to the positive part.

**Figure 4:**
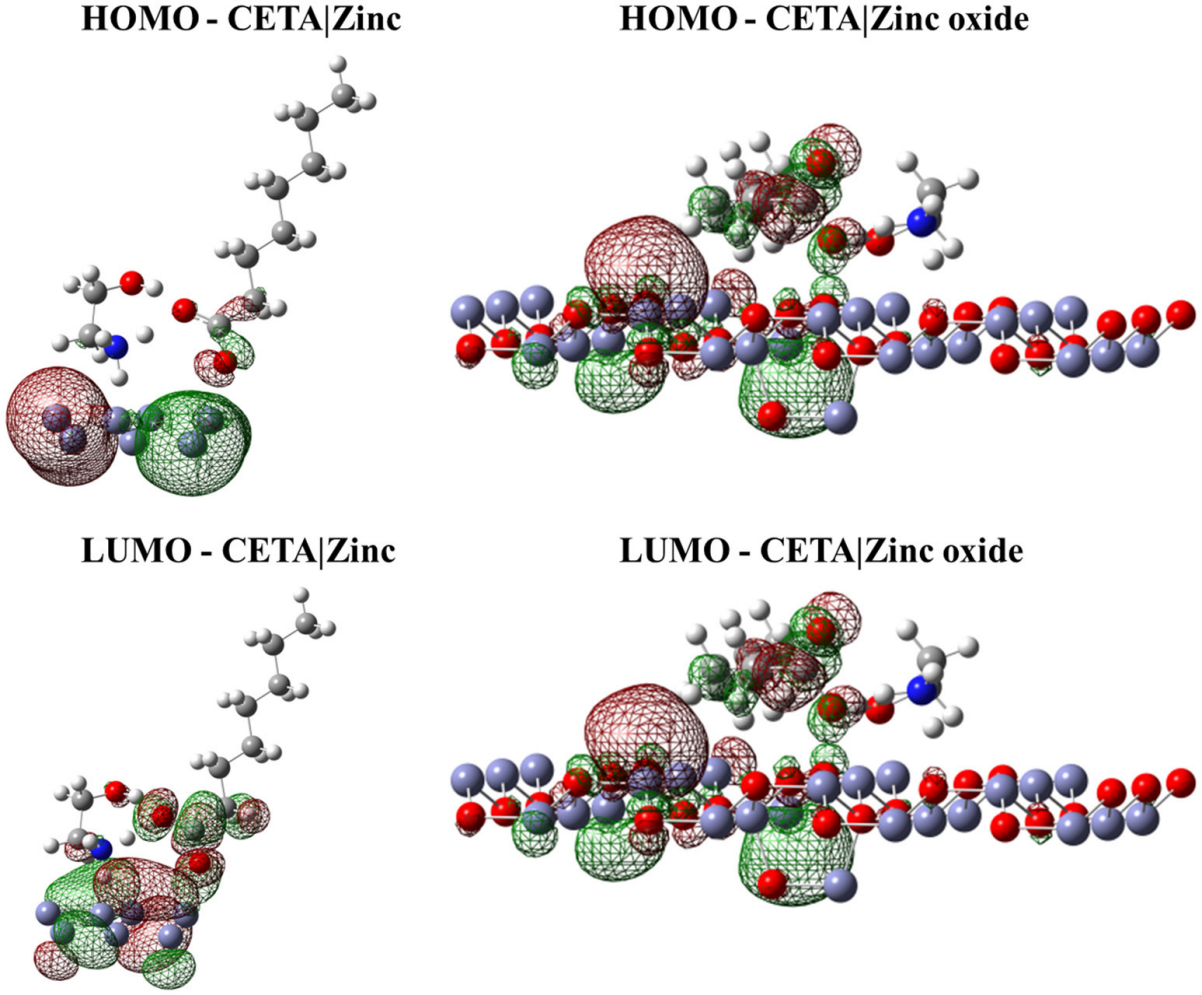
Isodensity surfaces (density value = 0.0001 A^−3^) of HOMO and LUMO orbitals of CETA molecule with the zinc and zinc oxide surfaces. The red color represents the negative part of the wave function and the green color corresponds to the positive part.

Despite the relevant information regarding the spatial arrangements of the VCI, it is also important to evaluate the interaction of the frontier orbital of these VCIs with the metal surface. These orbitals were calculated for the system in the minimum energy configuration and considering only the quantum region of the system. The isodensity surface of these orbitals with the respective surfaces is displayed in Figures 2–4.

Figures 2–4 illustrated that, for CCHA, CDCHA, and CETA salts, respectively, the HOMO and LUMO orbitals localized on the caprylate anion have shown strong shifting in direction to zinc and zinc oxide surfaces.

#### SCAN and analysis of Mullikan charges

**Figure 5:**
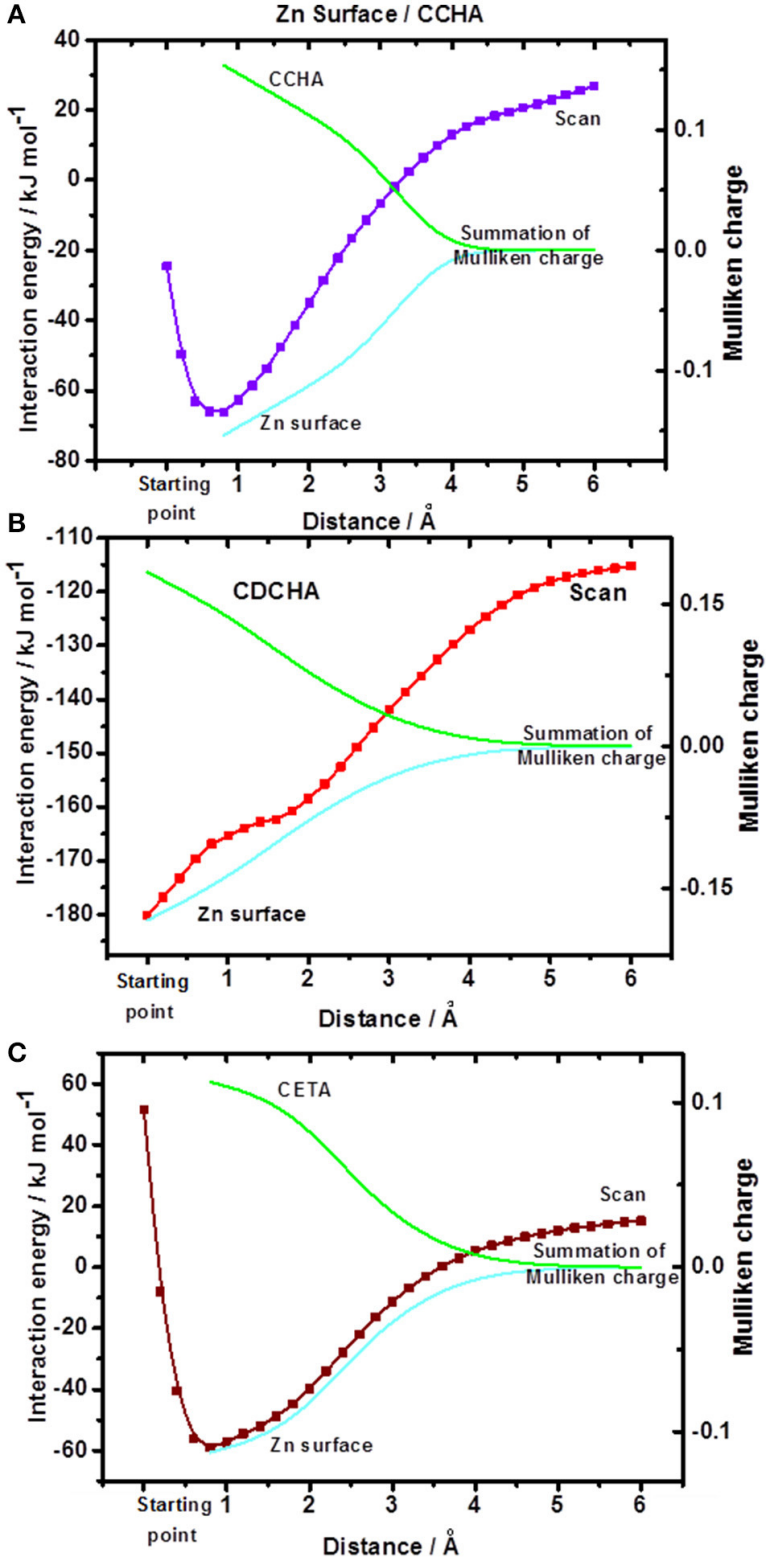
Potential energy surfaces and Mulliken charges: **(A)** CCHA-Zn surface; **(B)** CDCHA-Zn surface; **(C)** CETA-Zn surface. The starting point corresponds to the distance in the optimized system.

The energy performance of the system front to the different spatial arrangements of the inhibitor was observed by a process of departure from the equilibrium. For that, starting from the optimized system (the zinc/VCI surface), termed “starting point,” the separation of the metal surface from that of VCI was performed by varying the distance between the nitrogen atom and the nearest atom of zinc up to a distance of 6 Å, and then obtained the surface of potential energy (PES) (Frisch et al., 2009). Together, for the minimum energy interaction and sequentially at each 1 Å, the sum of the Mulliken charges of the zinc atoms and the sum of the Mulliken charges of the VCI atoms were obtained. Thus, the system is evaluated by the interaction energy of VCIs with the zinc surface, the overall energy gap, and their Mulliken charges at each point (Figure 5).

The potential energy surface (Figure 5) displays the VCI—zinc surface interaction energy as a function of the distance, as previously stated. The CDCHA caprylate salt had the highest interaction energy, around −180 kJ mol^−1^, followed by the CCHA and CETA salts, which presented interaction energy of −71 and −63 kJ mol^−1^, respectively. Based on the interaction energy values, it is noticed that the dicyclohexylamine salt showed greater interaction with the surface, followed by cyclohexylamine and ethanolamine salts.

For zinc oxide, only the interaction energy was calculated due to the excessive calculating cost comparing to metallic zinc. The following energy values were obtained: −71 kJ mol^−1^ (CCHA); −112 kJ mol^−1^ (CDCHA); and −125 kJ mol^−1^ (CETA). The highest energy value for CETA can be associated with the different ways of interaction between the amine and oxygen from the zinc oxide, and also due to strong ion-dipole interaction (dipole of ethanolamine vs. the zinc ion in the oxide. However, the high energy value for CETA cannot be directly related to an higher protection of zinc oxide surface against corrosion due to the formation of complex between zinc ions and ETA.

#### Agglutination of the VCI on the zinc surface

To understanding the interactions that occur between the VCI molecules (lateral interactions) attached on the surface, and those of VCI with the surface (vertical interaction), it was decided to expand the zinc surface area and then add VCI molecules step by step. Accordingly, VCI molecules were being added to the surface one by one, and the total energy of interaction, which includes the lateral and vertical interactions, has been obtained (Equation 2).

The minimum energy state for the system described herein reached a gradient of 10^−7^ where the optimization is considered to be converged. Possibly other spatial conformations could and would be achieved in the real environment, since this is a dynamic system where there are influence of temperature, solvents and others. Nevertheless, the theoretical results in this study are consistent with the experimental results shown below. In these calculations, the ONIOM methodology was used, but unlike the treatment already showed, the metal surface is treated on classical level (Molecular Mechanical, MM) (Rappé et al., 1992) and the molecules of the VCIs on quantum level (B3LYP/6-31g). Thus, in these calculations, the electronic effects of electron transfer between the surface and the VCI molecules are not evaluated by quantum calculations.

**Figure 6:**
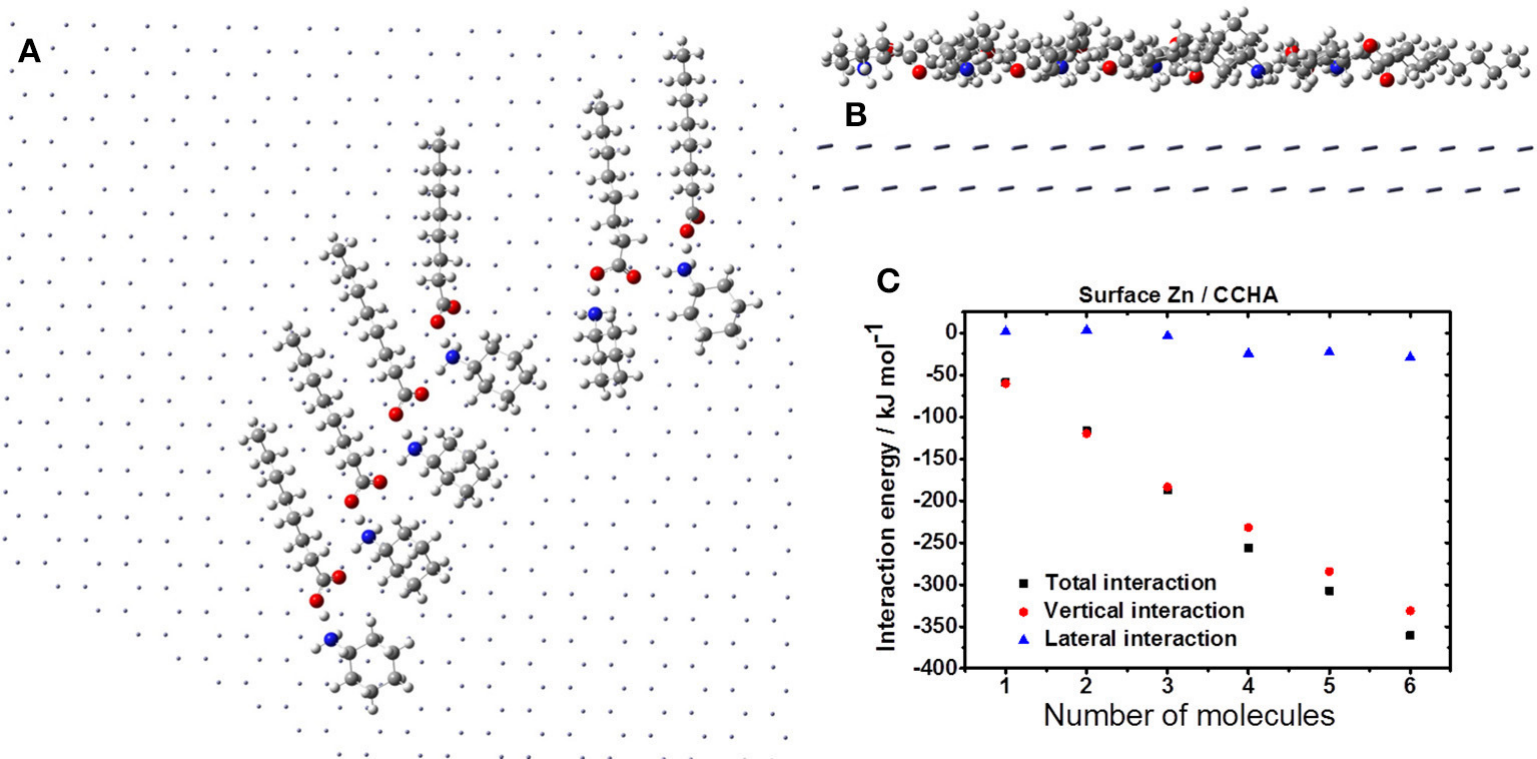
Formation of CCHA film on the zinc surface: horizontal **(A)** and vertical **(B)** views; **(C)** interaction energy.

**Figure 7:**
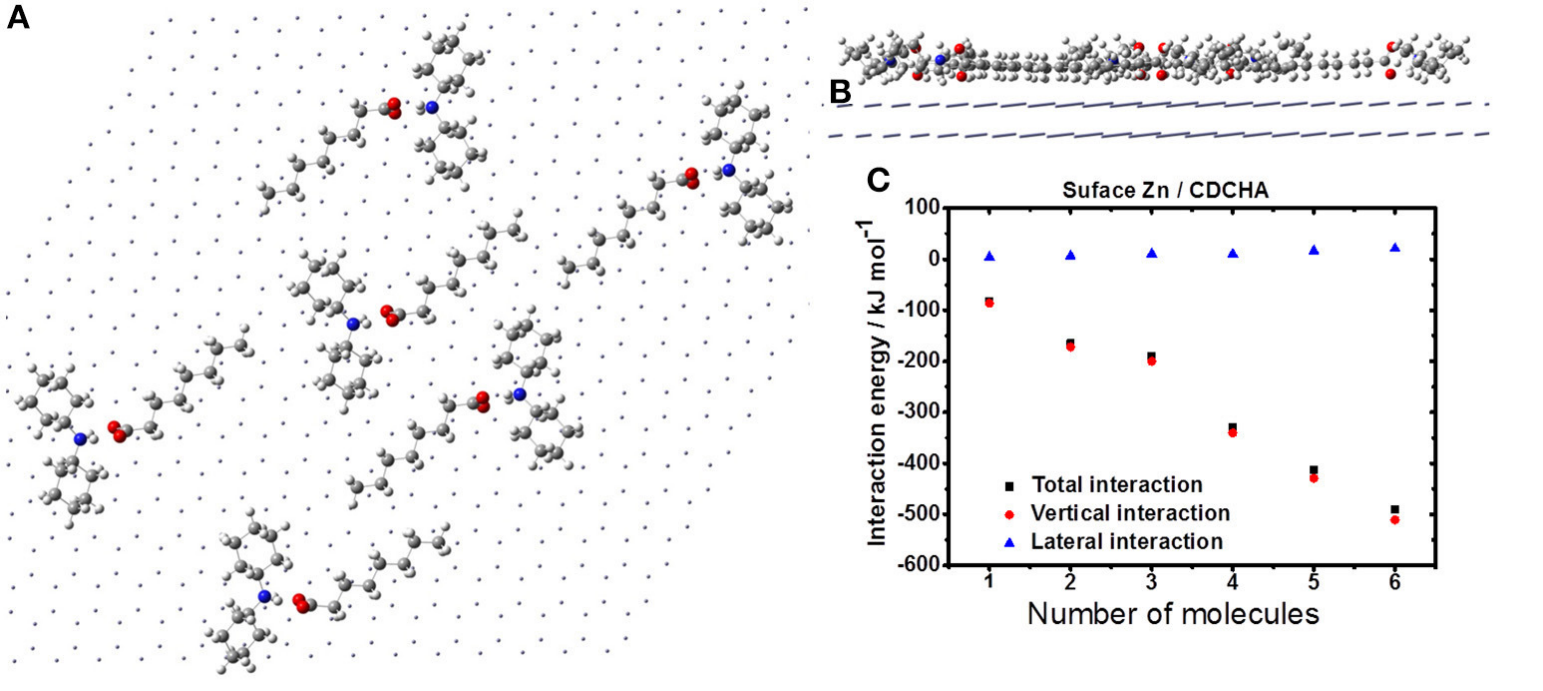
Formation of CDCHA film on the zinc surface: horizontal **(A)** and vertical **(B)** views; **(C)** interaction energy.

**Figure 8:**
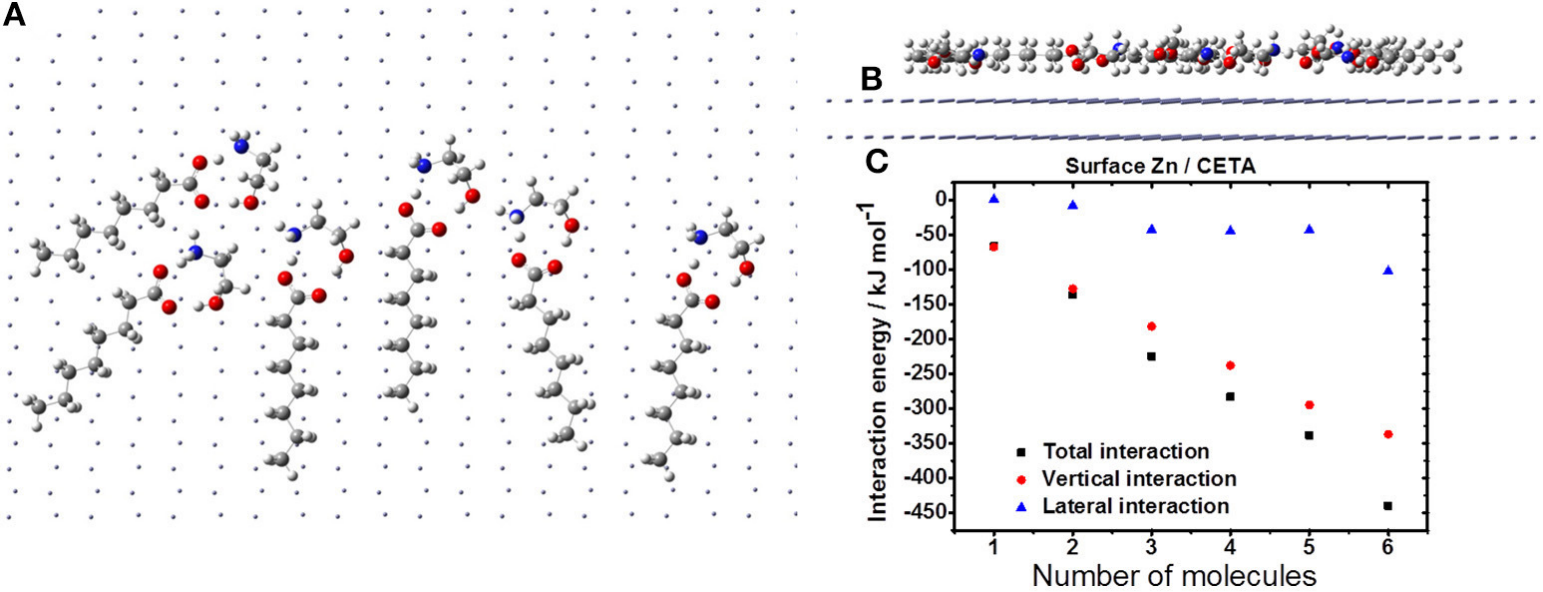
Formation of CETA film on the zinc surface: horizontal **(A)** and vertical **(B)** views; **(C)** interaction energy.

Figures 6–**8** show the agglutination of VCI CCHA, CDCHA and CETA, respectively. Figure 6 shows the agglutination of the CCHA on zinc surface. It is realized a parallel disposition of VCI molecules that is controlled by the H-bond and the aliphatic chain is parallel to the surface (Figure 6A). The surface coverage is enhanced by the parallel disposition in relation to the alkane cycle, and there is not great exposed surface area between the VCIs (Figure 6B). Considering the interaction energies, the H-bonds, when present, contribute for lateral interaction, which is demonstrated with 4 VCI molecules interacting each other (Figure 6C). The vertical interaction corresponds almost to the total interaction energy of the system, which means that the lateral interaction has low contribution.

Figure 7 displays the agglutination of the CDCHA on zinc surface. This VCI allows occupying a greater region on the metal surface due to its size and organized distribution (Figures 7A,B). Despite the disposition of the molecules the lateral interaction is destructive, thus each addition of CDCHA molecule the lateral interaction assumes a positive value (Figure 7C).

Figure 8 depicts the agglutination of the CETA on zinc surface. The molecules distribution is described by the H-bonds, and the aliphatic chain disposes parallel to the zinc surface (Figure 8A). Probably, the VCI molecules are going to assume a circular distribution due to the H-bonds, which may generate free space between the CETA molecules, exposing VCI-free zinc surface (Figure 8B), leading to a non-compact coating. Even considering the contribution of H-bonds to lateral interaction, the vertical interaction is more significant (Figure 8C). As consequence of the free space generated by the molecules distribution, lateral interaction, and strong dipole moment is expected the formation of complexes of ETA with zinc ions and the corrosion of zinc by chloride, resulting in the accumulation of porous products of corrosion to produce a poor protective film.

Although the explicit vertical interaction dominates the adsorption of various VCI on the zinc surface, it must be considered that the nature of the protection of these inhibitors on metal zinc involves oxidation/reduction processes, i.e., electrons are transferred between the compounds and the metal. Thus, the use of ONIOM method (B3LYP- 6-31g/MM) is limited to assess the vertical interaction, which is expected to express a substantial electronic effect to the corrosion inhibition process.

The observation is indeed interesting because it depicts that, although there is an extra lateral interaction, in the case of ETA, the possibility of interaction with two groups simultaneously on the same molecule (ETA is a hydrogen bond donor and acceptor) and the small space occupied by the ETA allow for a heterogeneous surface coverage, which is not necessarily efficient when considering blockage as the main mechanism of corrosion inhibition. The steric hindrance in CHA restrains the disposition to a situation where the salt bridges are shared among the units and dispersion interactions are favorable for the formation of a film of higher coverage. The DCHA is even more interesting: due to extra hindrance, the salt bridges can no longer interact closely, and the electrostatic interaction is more intense between the VCI and the metal surface, and the mechanism of inhibition can change.

### Experimental results

#### EIS

The open circuit potential (*E*_OCP_) for different concentrations of the studied VCIs and substrate was measured for 24 h of immersion. For short immersion times, the *E*_OCP_ decreased for all conditions studied and after around 9 h tended to stabilize. For each inhibitor, the *E*_OCP_ values slightly increased with the VCI concentration, and for 15 × 10^−3^ mol L^−1^ and after 24 h the following values were measured: CDCHA (−0.85 V); CCHA (−0.92 V); and CETA (−0.92 V). This result indicates that, mainly, the CDCHA molecule acts as an anodic inhibitor. For the substrate, the *E*_OCP_ values was −0.99 V after 24 h of immersion, indicating that the zinc surface is more active in the absence of inhibitors.

**Figure 9:**
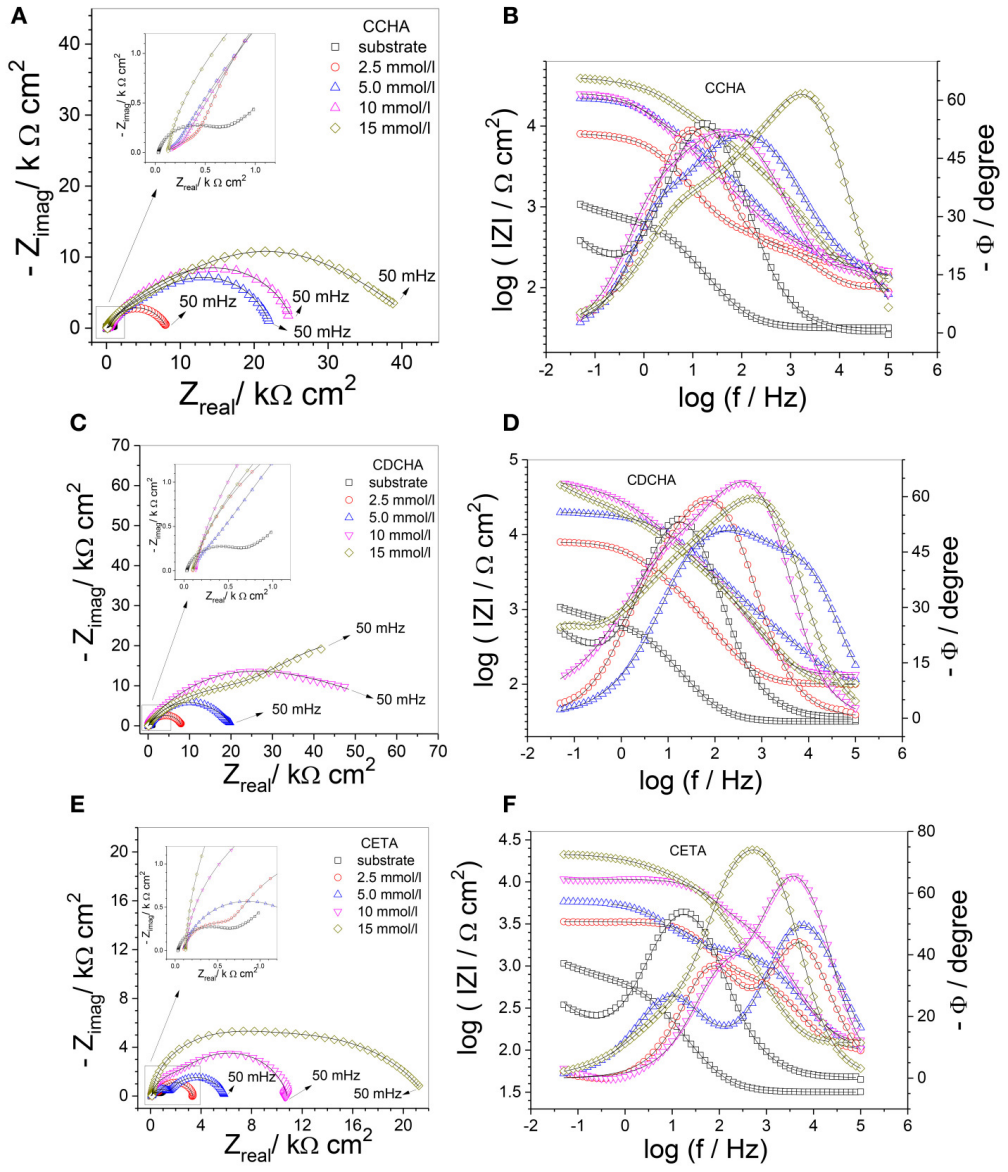
Complex plane diagrams **(A,C,E)** and Bode plots **(B,D,F)** of zinc in 0.1 mol L^−1^ NaCl in the absence and presence of different caprylate salts concentrations. Experimental data (symbol) and fitting (solid line). Inserts put in evidence the high frequency region and mainly the impedance response of the substrate.

Figure 9 shows experimental and fitted complex plane (Figures 9A,C,E) and Bode (Figures 9B,D,F) plots for zinc (substrate) in the absence and presence of inhibitors after 24 h of immersion in 0.1 mol L^−1^ NaCl aqueous solution at 25°C. For comparison, impedance diagrams of substrate were included in the diagrams of all VCIs.

The Nyquist diagram for zinc in the absence of VCI shows a capacitive arc at medium frequencies followed by Warburg characteristic associated to the diffusion inside the corrosion product layer (*W*_s_) which does not offer any protection to zinc. The Bode plots show two time constants, one at relatively high frequencies (≈15 Hz) that was related to the charge transfer resistance with the effect of the electrical double layer capacitance followed by a diffusion response inside of a non-protective corrosion product layer (Abd-El-Naby et al., 2012).

A zinc oxide and hydroxide layer naturally formed in air covers the zinc surface (Graedel, 1989) and may protect or not zinc against corrosion depending on pH. From pH 4 to 11 the corrosion of zinc is practically independent of pH, probably due to the occurrence of different cathodic processes from hydrogen evolution (at pH < 4) and the formation of unprotective surface oxides layer, meaning that the cathodic reaction changed from hydrogen evolution to oxygen reduction (Thomas et al., 2012). The corrosion of zinc in an aerated solution with pH around 7 occurs by a combination of anodic dissolution of zinc and cathodic reduction of oxygen (Aramaki, 2002),

(8) $$\begin{array}{l} \text{Zn }\to \text{Z}{\text{n}}^{2+}+2e^{-} \end{array}$$

(9) $$\begin{array}{l} {\text{O}}_{2}+2{\text{H}}_{2}\text{O}+4e^{-}\to 4\text{O}{\text{H}}^{-} \end{array}$$

The zinc hydroxide precipitated on the surface due to the low solubility and gradually changes to zinc oxide. Thus, a passive film of zinc hydroxides/oxides is formed on the electrode surface (D'Alkaine and Boucherit, 1997).

(10) $$\begin{array}{l} \text{Z}{\text{n}}^{2+}+2\text{O}{\text{H}}^{-}\;\to \text{Zn}{\left(\text{OH}\right)}_{2} \end{array}$$

(11) $$\begin{array}{l} \text{Zn}{\left(\text{OH}\right)}_{2}\;\to \text{ZnO}+\;{\text{H}}_{2}\text{O} \end{array}$$

In the presence of chloride, the zinc hydroxide reacts with chloride to form soluble complexes of zinc (Peulon, 1998), and, therefore, local dissolution of the passive film occurs, resulting in pitting (Guo et al., 1995), where the zinc oxidation and dissolution take place. Zinc oxide, in the presence of 0.1 mol L^−1^ NaCl, is the most stable phase, based on the thermodynamic, when the 7.7 ≤ pH ≤ 12 and from pH 4 to 11 the oxide/hydroxide-based film does not serve as an effective corrosion protection barrier, and for 0.5 mol L^−1^ NaCl solution simonkolleite (Zn_5_(OH)_8_Cl_2_) was detected (Thomas et al., 2012). In our work, the EDS analysis of the surface of zinc after 15 days in chloride solution (supplementary information, Figure S1) showed the presence of oxygen, chlorine, and zinc. The presence of these elements on the surface suggests the formation of zinc oxides and the corrosion product simonkolleite (Zn_5_(OH)_8_Cl_2_) (Autengruber et al., 2012).

The impedance spectra obtained for zinc after 24 h of immersion in 0.1 mol L^−1^ NaCl solution containing caprylates salts are also shown in Figure 9. The measured impedance is higher in the presence of VCI than in its absence, indicating that the inhibitor offers certain protection to the substrate, and the inhibition efficiency depends on the nature and concentration of the VCI. The Nyquist plots indicate the presence of at least two partially overlapped semicircles for CCHA and CETA (Figures 9A,E), and two well-separated semicircles for CDCHA (Figure 9C). It is also noted that the total real impedance (Z_real_) for the different VCIs increases in the following order: CETA < CCHA < CDCHA and with their concentration from 2.5 to 15 × 10^−3^ mol L^−1^.

For both CCHA and CDCHA the Bode phase angle *vs* log f plots (Figures 9B,D) suggest the presence of three time constants which were also required for fitting the data using an equivalent electrical circuit (EEC). For CETA (Figure 9F) only two time constants are observed in the Bode phase angle plots and an EEC with two time constants was required for fitting the experimental data (see below). For CCHA and CDCHA, the time constant at high frequency was attributed to the adsorbed inhibitor and appears at f > 100 Hz, the second time constant at around 10 Hz was attributed to the original oxide/hydroxide thin layer and the one at lower frequencies was related to the charge transfer process and capacitance of the electrical double layer. For CETA, the time constant at high frequencies was attributed to the adsorption and products of the reaction of ETA with zinc ions, and the time constant at lower frequency was attributed to the charge transfer process at the zinc/corrosion products layer interface. By comparing the Bode phase angle for both CCHA and CDCHA, it is clear that CDCHA shows higher capacitive behavior, and the time constant at higher frequency appears at f > 10^3^ Hz for CDCHA and f > 10^2^ Hz for CCHA. The maximum phase angle values remain almost constant for CDCHA and slightly shift to higher frequency values with the VCI concentrations for CCHA. For CETA, the maximum values for the time constant at high frequency slightly shift to lower frequency with the VCI concentrations, suggesting some attack to the zinc surface. The modulus of impedance |Z| at low frequency follows the order: CDCHA > CCHA > CETA for all studied VCI concentrations, and is 1 to 2 orders of magnitude higher for CDCHA than for the substrate.

**Figure 10:**
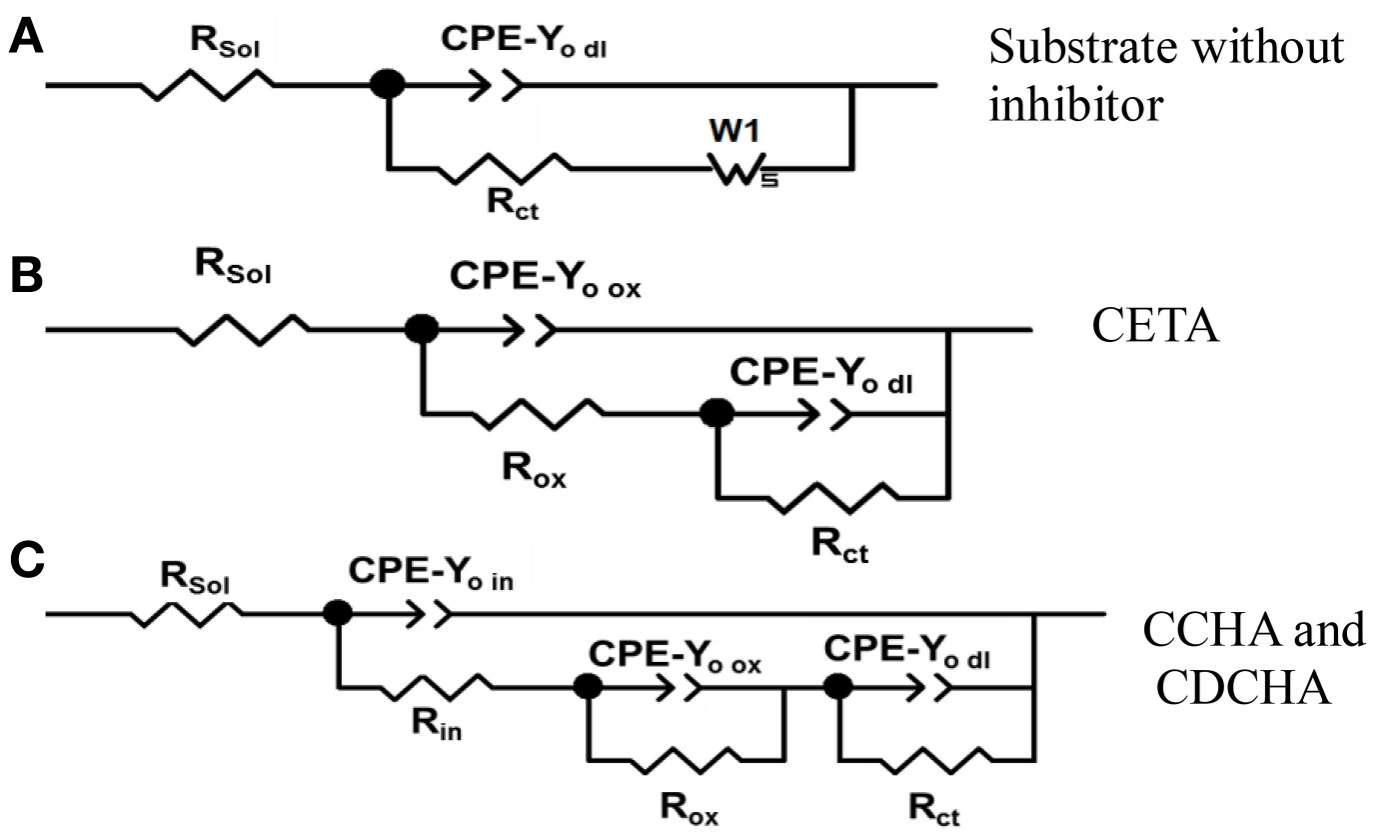
Equivalent electrical circuits used to adjust the experimental impedance data. **(A)** Substrate (Zinc) without inhibitors; **(B)** CETA (ethanol-ammonium caprylate); **(C)** CCHA (cyclohexyl-ammonium caprylate) and CDCHA (dicyclohexylammonium caprylate).

Figure 10 shows the electrical equivalent circuits (EECs) used to fit the impedance experimental data, where *R*_s_ corresponds to the solution resistance, *R*_in_, *R*_ox_, and *R*_ct_ are the resistances related to the response of the adsorbed inhibitor, oxides and hydroxides and/or eventually corrosion products adsorbed, and to the charge transfer resistance on the electrode/solution interface, respectively. CPE is the constant phase element related to the capacitance of the electrode with heterogeneous charge distribution at the surface, or different properties distributions on and through the films. The impedance of the constant phase element can be represented by Orazem and Tribollet (2008):

(12) $$\begin{array}{l} Z_{CPE}=Y_{\text{o}}^{-1}\;{\left(\text{j}\omega \right)}^{-\text{n}} \end{array}$$

where *j* = (−1)^1/2^, ω is the angular frequency (rad s^−1^), *Y*_*o*_ is associated to the capacitance and *n* is the exponent that can be related to the heterogeneity degree of the surface or to the non-ideal behavior of the properties distribution through the adsorbed layer and film. For *n* = 0, *CPE* represents a resistor (*R* = *Y*_*o*_^−1^), *n* = 1 means that the capacitive behavior is represented by an ideal capacitor (*C* = *Y*_*o*_) and a *n* = 0.5 corresponds to a diffusion process at low frequencies (Warburg) or a porous electrode at high frequencies (Orazem and Tribollet, 2008). The CPE sub-indexes in, ox, and dl represent inhibitor, oxide, and electrical double layer, respectively. *W*_*s*_ represents the diffusion of O_2_ or Zn^2+^ ions through the adsorbed film and corrosion products layer.

To choose equivalent electrical circuit model to analyze the impedance data the following considerations were done. According to the impedance diagram above described for the substrate in the absence of inhibitor, the EEC (Figure 10A) fitted well the impedance results. The action of chloride on the substrate surface deteriorated the natural compact zinc oxide layer, facilitating the charge transfer process. This process is characterized by the *CPE-Y*_o dl_ constant phase element in parallel with the charge transfer resistance *R*_ct_ which is in series with a Warburg element represented by *W*_s_ that accounts for the diffusion of zinc ions and/or oxygen through the porous and non-protective corrosion products layer.

When VCIs are in solution, the presence of the adsorbed layer on zinc and/or zinc oxide surface is evident by comparing the impedance diagrams for zinc (Figure 9) in the presence and absence of inhibitors. The EDS analysis (Figure S1) confirmed the presence of oxygen probably associated with the oxide/hydroxide layer and/or some product like simonkolleite on the zinc surface. For CCHA and CDCHA, different from zinc substrate and CETA, the natural and compact zinc oxide thin film is supposed to be still present during the time course of the electrochemical experiment in 0.1 mol L^−1^ NaCl solution. The adsorption of CCHA and CDCHA should prevent a rapid attack from chloride to the oxide layer and zinc. The charge transfer process occurs through the oxidation of zinc in defects of the oxide film. Therefore, the electrode/solution interface may be represented as an inhibitor layer on zinc oxide layer in contact with the chloride solution, and the impedance data were well-fitted using the equivalent circuit of Figure 10C. Thus, the outer adsorbed layer was characterized by a parallel combination of a layer constant phase element *CPE*_in_ and a layer resistance *R*_in_. The inner oxide compact layer and the corroding interface were disposed in series with *R*_in_. The compact layer is also a parallel combination of a layer constant phase element *CPE-Y*_o ox_ and layer resistance *R*_ox_ that represents the resistance of the solution inside the pores of this layer, and the corroding interface is characterized by a parallel combination of an electrical double layer constant phase element *CPE-Y*_o dl_ and a charge transfer resistance *R*_ct_.

For CETA, the impedance data were adjusted using the EEC of Figure 10B, which is a parallel combination of two CPE//R sub-circuits: one representing the adsorbed VCI together the unprotective corrosion product layer and the other the charge transfer process. The less protection of this layer is due to the nature of interaction between ETA and zinc species. The strong interaction of ETA with zinc ions can be due to the highest dipolar moment of CETA (Table 1) that results in the formation of zinc complexes, log K = 2.41 for ionic strength 0.1 mol L^−1^ (Martell et al., 1997) and other zinc chloride complexes around pH 7 (Thomas et al., 2012). These complexes are relatively soluble and the competition between ETA adsorption and complexes dissolution exposes zinc to chloride decreasing the impedance and consequently the inhibitor efficiency. Therefore, the unprotective character of the adsorbed and corrosion product layer formed on zinc surface allowed to use the EEC of Figure 10B. There is no information about the complex formation between zinc ions and CCHA or CDCHA.

The presence of CHA, DCHA, caprylate, zinc oxide, and simonkoleite were detected by Raman studies of the zinc surface after 21 days of immersion in 0.1 mol L^−1^ NaCl solution containing 15 × 10^−3^ mol L^−1^ of VCI (Figure S2 and Table S1, supplementary information) indicating that the VCI is adsorbed on the surface and that ethanolamine was not detected, probably, due to the complex formation with zinc ions.

Table 2 shows the parameters obtained by the adjustment of the experimental data using EECs. The low error values associated to each parameter and a value of c^2^ around 10^−4^–10^−5^ indicates a good fitting, that suggests these models allow a possible interpretation of experimental data.

**Table 2:** EEC parameters obtained from EIS diagrams using electrical equivalent circuits.

**C_in._(10^−3^ mol L^−1^)**		***E*_OCP_(V)**	**EEC**	***R*_*sol*_(Ω cm^2^)**	***CPE-Y*_o in_(S s^*n*^ cm^−2^)**	***n_*in*_***	***R*_in_(Ω cm^2^)**	***CPE-Y*_o ox_(S s^n^ cm^−2^)**	***n_*ox*_***	***R_ox_*(Ω cm^2^)**	***CPE-Y*_o dl_(S s^n^ cm^−2^)**	***n_*dl*_***	***R_ct_*(Ω cm^2^)**	**EI (%)**	**X^2^**
CCHA	2.5	−0.96	3	88 (4%)	1 10^−5^(4%)	0.5 (16%)	123 (1%)	1.7 10^−5^(7%)	0.63 (1.1%)	329 (0.38%)	1.3 10^−5^(1%)	0.89 (0.3%)	7,712 (0.2%)	92.2	4.2 10^−5^
	5	−0.95	3	121 (0.3%)	1.2 10^−6^(1%)	0.75	144 (1%)	6.5 10^−6^(1%)	0.67 (0.17%)	8,813 (1.6%)	1.4 10^−5^(1%)	0.89 (0.5%)	13,190 (1%)	97.1	2.6 10^−5^
	10	−0.92	3	141 (0.8%)	4 10^−7^(20%)	0.83 (2%)	127 (3%)	1.2 10^−5^(3.8%)	0.67 (0.3%)	8,063 (4.7%)	1.6 10^−5^(2.8%)	0.86 (0.8%)	17,224 (2.7%)	97.5	5.0 10^−5^
	15	−0.92	3	125 (0.5%)	2.8 10^−7^(5%)	0.9 (0.5%)	2,555 (9%)	7.7 10^−6^(7%)	0.5 (3%)	34,322 (31%)	1.5 10^−5^(2.8%)	0.99 (8%)	5,448 (31%)	98.5	1.8 10^−4^
CDCHA	^*^2.5	−0.96	2	99 (0.6%)	—	—	—	3.1 10^−5^(2%)	0.72 (1%)	4,200 (2%)	9.3 10^−6^(1%)	0.83 (0.1%)	3,809 (1.9%)	92.1	8.4 10^−5^
	5	−0.95	3	102 (0.7%)	4.3 10^−7^(12%)	0.83 (1%)	549 (7%)	4.3 10^−6^(12%)	0.62 (0.5%)	17,980 (1%)	1.3 10^−5^(4%)	0.95-	1,267 (1%)	96.8	6.2 10^−5^
	10	−0.94	3	133 (0.2%)	6.7 10^−7^(5%)	0.93 (0.4%)	1,702 (16%)	1.7 10^−5^(22%)	0.42 (5%)	50,052 (8.7%)	1.2 10^−5^(13%)	0.74 (6%)	13,884 (33%)	99.0	7.8 10^−5^
	15	−0.85	3	98 (0.1%)	9 10^−7^(1%)	0.87 (0.1%)	1,642 (3%)	1.2 10^−5^(1%)	0.5 (0.5%)	33,332 (2%)	1.1 10^−4^(3.8%)	0.82 (2%)	45,540 (4%)	99.2	1.0 10^−5^
CETA	2.5	−0.98	2	99 (0.5%)	—	—	—	4.2 10^−7^(4.2%)	0.89 (0.5%)	703 (1.2%)	4.0 10^−6^(2.8%)	0.87 (0.6%)	2,560 (0.6%)	80.5	5.0 10^−4^
	5	−0.93	2	101 (0.5%)	—	—	—	3.5 10^−7^(2.6%)	0.84 (0.3%)	1,417 (0.6%)	2.0 10^−5^(2.2%)	0.76 (0.8%)	4,380 (1.2%)	89.0	4.7 10^−4^
	10	−0.93	2	125 (0.5%)	—	—	—	9.2 10^−8^(3.2%)	0.96 (0.3%)	3,289 (1.9%)	1.1 10^−6^(3.8%)	0.81 (0.9%)	7,400 (1.1%)	94.1	1.2 10^−4^
	15	−0.92	2	121 (0.2%)	—	—	—	3.6 10^−7^(1.5%)	0.96 (0.2%)	8,648 (2.4%)	1.2 10^−5^(4.9%)	0.59 (3.3%)	13,134 (4.0%)	97.1	2.2 10^−4^
**C_*in*._(10^−3^ mol L^−1^)**		***E*_*OCP*_(V)**	**EEC**	***R*_*sol*_(Ω cm^2^)**	***CPE-Y*_*o dl*_(S s^*n*^ cm^−2^)**	***CPE-n dl***	***R*_*ct*_(Ω cm^2^)**	***Ws-T* (s cm^−2^)**	***Ws-P***	***Ws-R* (Ω cm^2^)**				**EI (%)**	**X^2^**
Substrate without inhibitor		−0.99	1	32 (0.2%)	1.3 10^−4^(1.0%)	0.84 (0.2%)	635 (0.1%)	36 (25%)	0.53 (2.0%)	2,101 (14%)	—	—	—	—	3.6 10^−4^

* *For this CDCHA concentration the fitting with EEC of Figure 10C produced high errors for individual parameters, suggesting that another EEC should be used and then the EEC of Figure 10B was used*.

In the presence of caprylate salts, *CPE-Y*_*o*_ parameters decrease two orders of magnitude, which indicates that the inhibitors were absorbed on the zinc surface. The constant phase element is also directly related to the capacitance of the electric double layer (*C*_dl_) (Brug et al., 1984) and with the adsorption inhibitors the thickness of the double layer increases and hence the *C*_*dl*_ decreases. This trend agrees with the model of Helmholtz given by the following equation (Orazem and Tribollet, 2008):

(13) $$\begin{array}{l} C_{\text{dl}}=\;\frac{\varepsilon \;\varepsilon_{\text{o}}}{\text{d}} \end{array}$$

in which *d* is the thickness of the adsorbed layer, ε is the dielectric constant of the adsorbed layer and ε_o_ is the permittivity of free space. It is also noted that the *n*_ox_ is around 0.5 for CCHA and DCHA, suggesting the existence of pores in the oxide layer originated from the attack to the defects of the oxide. The resistance *R*_ct_ increases with the increase of the inhibitor concentration mainly for CDCHA and CCHA, being higher for DCCHA.

The impedance data also show that all the compounds have good inhibiting properties. The Inhibition efficiency, EI (%), is calculated from the polarization resistance (*R*_p_). In this case, the polarization resistance (*R*_p_) can be considered the sum of the resistances related to each time constants (Cai, 1996), according to Equation (14):

(14) $$\begin{array}{l} R_{\text{p}}=R_{\text{ct}}+R_{\text{ox}}+R_{\text{in}} \end{array}$$

and the EI (%) using Equation (15).

(15) $$\begin{array}{l} \text{EI}\left(\%\right)=\left(1-\frac{R_{\text{p}}}{R_{\text{p}}^{*}}\right)100 \end{array}$$

where *R*_p_ and $R_{\text{p}}^{*}$ are the polarization resistances in the absence and presence of inhibitor, respectively. *R*_p_ values were obtained using (Equation 14), where the different *R* values correspond to the data obtained from the equivalent circuit. The inhibition efficiency increases as the concentration of inhibitor increases (Table 2) and CDCHA showed the best performance followed by CCHA and CETA, respectively. The better performance of CDCHA can be explained due to the lower solubility of this amine, the higher hydrophobic character, and steric effect due to the volume of the molecule, as indicated in Figure 7.

#### Adsorption isotherms analysis

The adsorption mechanism of the corrosion inhibitor can be analyzed by the adsorption isotherm models, which are able to provide information about metal-inhibitor interactions. Different factors are involved in the adsorption process such as electronic characteristics of the inhibitor, nature of the metal surface, temperature, steric effects, and the various degrees of the surface sites activity (El-Awady et al., 1992). According to Ateya and El-Anadouli (1984) and El-Awady et al. (1992), the adsorption of an inhibitor on the metallic surface is considered as a process of replacing the water molecules adsorbed on the electrode surface by the inhibitor present in the solution or environment:

(16) $$\begin{array}{l} \text{Inhibitor}{\;}_{\left(\text{solution}\right)}+\text{x}\;{\text{H}}_{2}O_{\left(\text{ads}\right)}\;↔\text{ Inhibito}{\text{r}}_{\left(\text{ads}\right)}+\text{x }{\text{H}}_{2}O_{\text{solution}} \end{array}$$

where x is called size factor. Physically, the x is defined as the number of solvent molecules substituted by one adsorbed molecule of the inhibitor. The quantitative value of x depends on the model used to describe the structure of the adsorption layer. For models which consider the formation of a monolayer, x is defined by Trasatti (1992):

(17) $$\begin{array}{l} \text{x}=\;\frac{A_{\text{ocup}}^{*}}{{\text{A}}_{\text{ocup}}} \end{array}$$

where ${\text{A}}_{\text{ocup}}^{*}$ and A_ocup_ are the surface areas occupied by the inhibitor and water molecules, respectively.

Adsorption isotherms can describe the adsorption equilibrium and, generally all isotherm models assume the form (Kern and Landolt, 2001):

(18) $$\begin{array}{l} \text{f}\left(\theta ,\text{ x}\right){\text{e}}^{-2\text{aθ}}=\text{kC} \end{array}$$

were k is the adsorpotion equilibrium constant, C is the inhibitor concentration, θ is the surface coverage and *a* is the molecular interaction parameter that depends on the molecular interactions at the adsorption layer (Frumkin isotherm) or surface heterogeneity parameter (Temkin isotherm). Often it is used isotherms with *x* or *a* parameter explicit, but hardly both. Langmuir isotherm is the most common isotherm used (Damaskin et al., 1971):

(19) $$\begin{array}{l} \frac{\theta }{\left(1-\theta \right)}=\text{kC} \end{array}$$

and in the logarithm form:

(20) $$\begin{array}{l} \log\left(\frac{\theta }{1-\theta }\right)=\log\text{k}+\log\text{C} \end{array}$$

The Langmuir model assumes that the inhibitor will replace only one water molecule to adsorb (*x* = 1) on the metallic surface, there are no lateral interactions between the adsorbed molecules, and the surface is homogeneous (*a* = 0), it means that all active sites has the same energy.

Temkin isotherm is often used to describe heterogeneous surfaces, and is given by the equation (Bastidas et al., 2005b):

(21) $$\begin{array}{l} {\text{e}}^{-2\text{aθ}}\;=\text{kC} \end{array}$$

and in the logarithm form:

(22) $$\begin{array}{l} \theta =-\frac{1}{2\text{a}}\log\text{C}-\frac{\log\text{k}}{2\text{a}} \end{array}$$

where the heterogeneity parameter of the surface, *a* must be negative. *a* < 0 indicates that the adsorption sites have different energies. However, this isotherm does not consider the size of inhibitors (x = 1) and neither the lateral interactions.

When the lateral interactions are taken into consideration, Frumkin isotherm is the most common (Damaskin et al., 1971):

(23) $$\begin{array}{l} \frac{\theta }{\left(1-\theta \right)}e^{-2a\theta }\;=kC \end{array}$$

and in the logarithm form:

(24) $$\begin{array}{l} \log\left(\frac{\theta }{1-\theta }\right)-\log C=\log k+2a\theta \end{array}$$

where the lateral interaction parameter *a* may be positive or negative. *a* > 0 indicates an increase of the adsorption energy as θ increases due to the attraction between molecules, and *a* < 0 means repulsion between the molecules. However, Frumkin isotherm considers a homogeneous surface and disregards the size of inhibitors (x = 1).

Dhar-Flory-Huggins' isotherm has been used to describe the adsorption process for large molecule, where x > 1 (Dhar et al., 1973):

(25) $$\begin{array}{l} \left(\frac{\theta }{{\text{e}}^{\left(\text{x}-1\right)}{\left(1-\theta \right)}^{\text{x}}}\right)=\text{kC} \end{array}$$

and in the logarithm form:

(26) $$\begin{array}{l} \log\theta -\left(\left(\text{x}-1\right)+\text{x}\log\left(1-\theta \right)\right)=\log\text{k}+\log\text{C} \end{array}$$

In this isotherm, the size parameter x was previously calculated using Equation (17), and data from Table 1, where the area occupied by the water A_ocup_ was calculated to be 11.1 Å^2^ per molecule.

The surface coverage (θ) was calculated based on the polarization resistance (*R*_*p*_) obtained from the data of Table 2.

(27) $$\begin{array}{l} \theta \;=\;1-\;\frac{R_{\text{p}}}{R_{\text{p}}^{*}} \end{array}$$

where $R_{\text{p}}^{*}\;$ and *R*_p_ represent the polarization resistance in the presence and absence of inhibitor, respectively.

The adsorption equilibrium constant *K* is related to the standard Gibbs energy of adsorption ($\Delta G_{ads}^{{}^{\circ}}$) (Ateya and El-Anadouli, 1984):

(28) $$\begin{array}{l} \Delta {\text{G}}_{\text{ads}}^{\text{o}}=-\text{RT}\ln\;\left(55.5\text{K}\right) \end{array}$$

It is well-stablished that a $\Delta G_{\text{ads}}^{{}^{\circ}}$ < −30 kJ mol^−1^ corresponds to the so called chemical adsorption (chemisorption) while a $\Delta G_{\text{ads}}^{{}^{\circ}}$ > −30 kJ mol^−1^ is related to physical adsorption (physisorption) (Macedo et al., 2012).

All adsorption isotherms were evaluated considering their theoretical basis, correlated with theoretical data and with their linearity, i.e., the isotherm that presents closest r^2^ to 1 may be that one that best applies to the system. The isotherm that better represented the results obtained for all VCIs was the Dhar-Flory-Huggins (Figure S3, supplementary information). The parameters obtained from the adsorption isotherm are in Table 3.

**Table 3:** Parameters obtained from the Dhar-Flory-Huggins adsorption isotherm.

**Isotherm**	**Parameters**	**CCHA**	**CDCHA**	**CETA**
Dhar-Flory-Huggins	*K*_ads_	7.9 10^13^	3.2 10^25^	6.3 10^12^
	$\Delta G_{\text{ads}}^{{}^{\circ}}$(kJ mol^−1^)	−89.2	−155.4	−83.0
	x^*^	5.5	6.7	5.0
	r^2^	0.91	0.98	0.97

* *The x parameter was previously calculated*.

**Figure 11:**
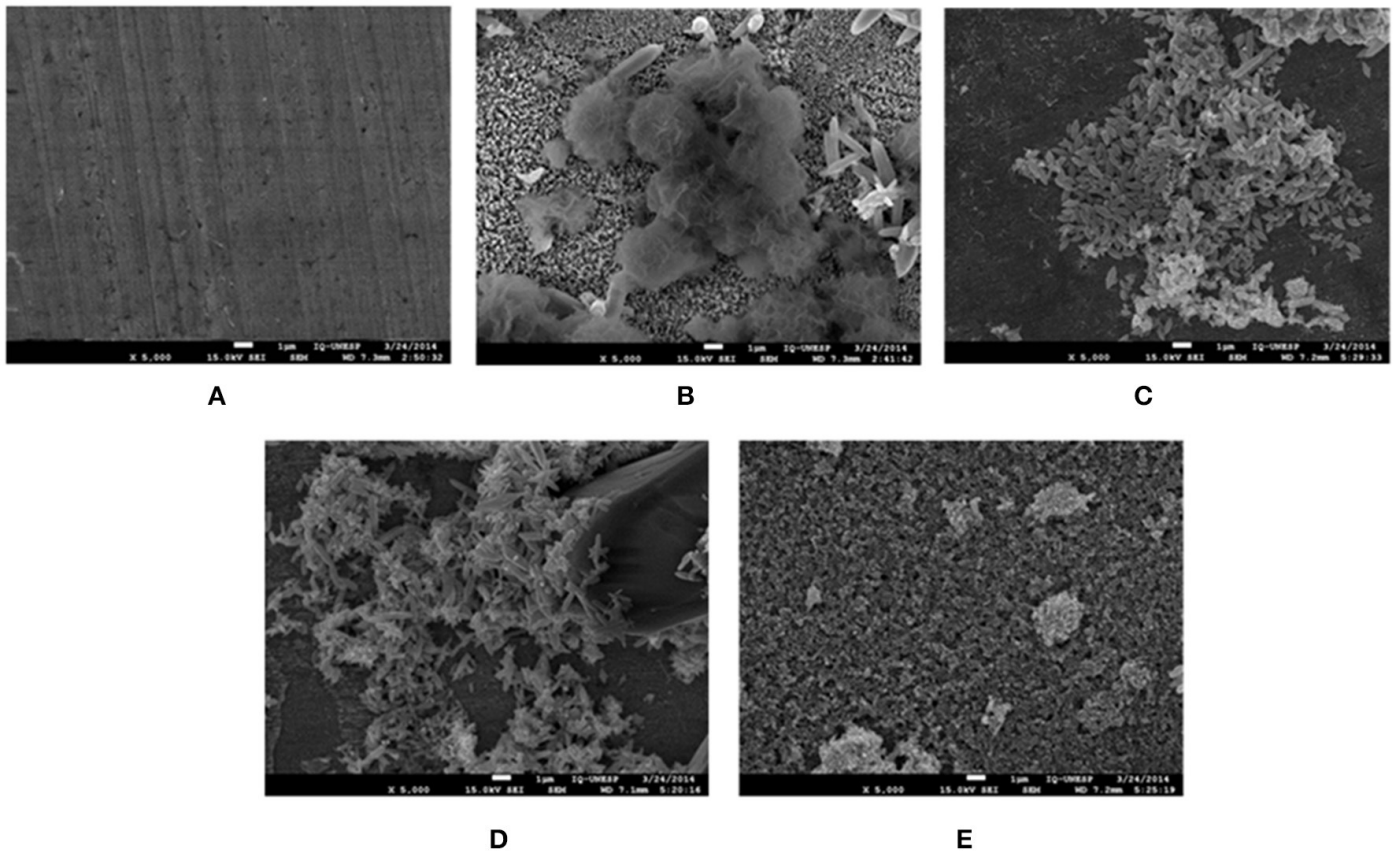
SEM micrographs of zinc surface before **(A)** and after 15 days of immersion in 0.1 mol L^−^^1^ NaCl solution in the absence **(B)** and presence of 5 × 10^−^^3^ mol l^−^^1^ VCI: **(C)** CCHA; **(D)** CDCHA; **(E)** CETA.

**Figure 12:**
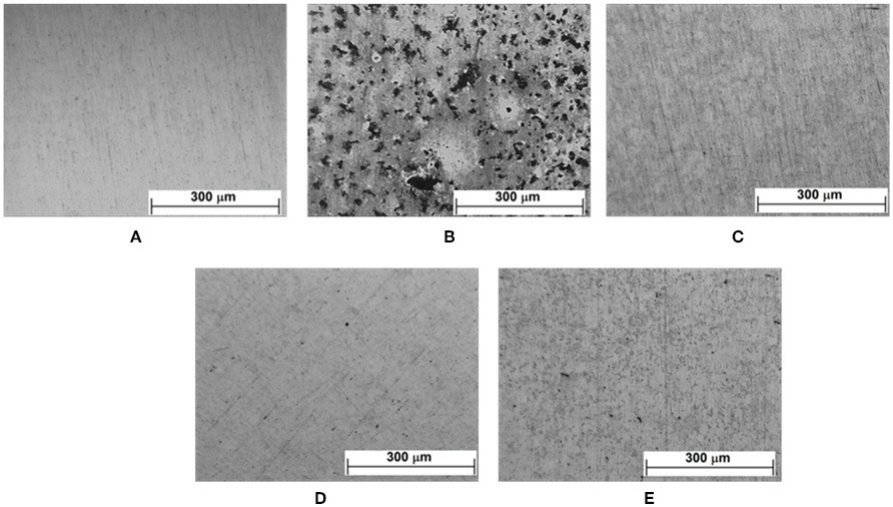
Optical micrographs of the zinc surface after conditioning in a humid chamber for 7 days in the absence and presence of VCIs: **(A)** after polishing; **(B)** without inhibitor; with inhibitor after 7 days of immersion **(C)** CCHA; **(D)** CDCHA; **(E)** CETA.

The CCHA and CDCHA salts followed the Dhar-Flory-Huggins isotherm while CETA salts gave good results also with Temkin's model. Δ*G*°_ads_ value was more negative for CDCHA. The theoretical calculations indicated that all studied VCIs presents strong vertical interactions (Figures 6C, 7C, 8C) and CCHA (Figure 6C) also shows very low lateral interaction while CDCHA shows no lateral interaction, and CETA shows the strongest lateral interaction among these VCIs. So, according to theoretical predictions, the Frumkin isotherm could not describe the behavior of CCHA and CDCHA. The Frumkin's model also did not described the behavior of CETA even considering that CETA presents lateral interaction (Figure 8C), probably because many other factors are involved in CETA-zinc surface interaction. The bad fitting to the Frumkin isotherm can be attributed to the high contribution of vertical interaction which corresponds almost to the total interaction energy. The fact that CETA follows also the Temkin's model may indicate that the surface of zinc becomes very heterogeneous due to the attack of ETA and chloride to zinc (see Figures 11D, 12E). A possible explanation that all these VCIs follows the Dhar-Flory-Huggins isotherm is that all have a size factor higher than 5.

In the Dhar-Flory-Huggins' model where Δ*G*°_ads_ is corrected to the size factor (x), experimental data of all caprylates inhibitors fitted well the model. Again, the CDCHA showed the most negative adsorption energy followed by CCHA and CETA, respectively. In general, the Dhar-Flory-Huggins' isotherm describes well the adsorption process, since x > 1 for all compounds. In this model, adsorption energies are more negative for salts derived from amines with greater size. Δ*G*°_ads_ more negative than −30 kJ mol^−1^ indicate chemical adsorption for all molecules in the following order: CDCHA < CCHA < CETA. Therefore, the analysis using different adsorption isotherms showed that the size of the molecule develops a great importance in the adsorption process of inhibitors. However, it is noticed that the isotherm which considers the size factor ignores lateral interactions that can introduce some deviation from the real behavior.

### SEM observations

Figure 11 displays the SEM micrographs of the zinc surface obtained after 15 days of immersion in 0.1 mol L^−^^1^ NaCl aqueous solution in the absence and presence of VCI. Deposits are clearly observed on the zinc surface and are different from one system to another.

Despite the differences in the SEM images, the EDS (Figure S1, supplementary information) detected the presence of zinc, oxygen, and chlorine elements in the deposits in the absence of inhibitor and oxygen and zinc in the presence. The presence of these elements on the surface suggests that in the presence of VCIs mainly zinc oxides are formed and in the absence of VCIs can be also formed the corrosion product simonkolleite (Zn_5_(OH)_8_Cl_2_) (Autengruber et al., 2012).

### Accelerated corrosion tests/moist chamber

After conditioning the samples for 7 days in a humidified chamber, optical micrographs of the surface were obtained (Figure 12). The attack on the surface was VCI-type dependent. Despite the presence of corrosion spots, it can be seen (Figures 12C–E) that the vapors of the caprylate salts provided some protection to the surface in the following order: CDCHA > CCHA > CETA. In a previous study (Teixeira et al., 2015), the presence of vapors of CHA, DCHA and ETA amines also modified the zinc surface: DCHA protected the surface and no corrosion was observed; CHA showed partial protection with the presence of corrosion spots; ETA oxidized all the zinc surface (Teixeira et al., 2015). The poor performance of CETA is related to the complexes formation with ETA and the free space suggested from theoretical calculations (Figure 8) due to the ETA the strong lateral interactions and molecules distribution on the zinc surface.

### Correlation between experimental and theoretical results

The correlation between experimental and theoretical data was done in two ways: by comparing the adsorption energies or the polarization resistances with the physicochemical properties of the inhibitor.

**Figure 13:**
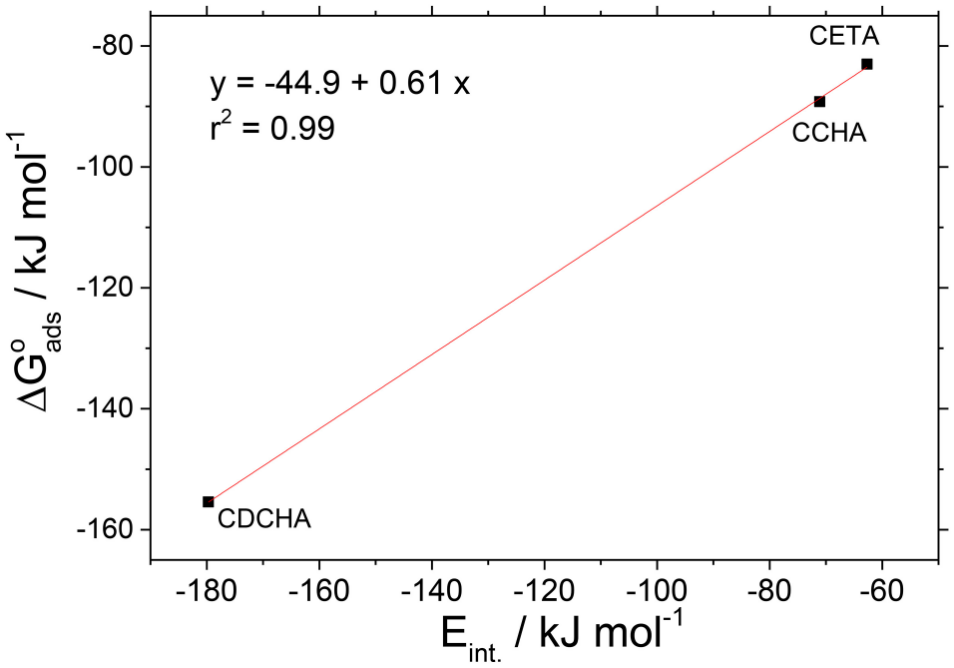
Correlation between the adsorption energies (experimental) and the interaction energies (theoretical) for VCI salts.

The experimental adsorption energies obtained from adsorption isotherms were correlated with those obtained from theoretical calculations considering the state of minimum energy. The isotherms used to these correlations were the Dhar-Flory-Huggins for caprylates salts. Figure 13 shows the correlation between experimental adsorption energies, Δ*G*°_ads_ and theoretical interaction energies, *E*_int_. The correlation coefficient (*r*^2^ = 0.99) can be considered acceptable since the adsorption models applied to the experimental data do not consider together the lateral interactions, the size factor and heterogeneity of the surface.

The efficiency of the caprylate salt was related to the respective quantum descriptors. It was then possible to obtain a mathematical correlation between the polarization resistance values of each inhibitor at different concentrations and the chemical structure of the salts to obtain a functional relationship between inhibition efficiency and descriptors. Thus, we used the results of EIS, the occupied area values (A_ocup_) and the energies of the frontier orbitals (HOMO e LUMO) (Bentiss et al., 2003). Like this, a correlation between experimental and theoretical data for caprylate salts was obtained by applying the multiple linear resistance model (Bentiss et al., 2003; Outirite et al., 2010) (Equation 29). This relationship allows to minimize experimental variations of different systems.

(29) $$\begin{array}{l} R_{\text{p}}=\;\sum_{i}\left({\text{APar}}_{1}+{\text{BPar}}_{2}+\;\ldots +{\text{CP}}_{\text{n}}\right)\;C_{\text{in}} \end{array}$$

where *R*_p_ is the polarization resistance, A, B, and C are linear regression coefficients obtained from chemical parameters (*P*_ar_) and *C*_in_ in mol L^−1^ is referred to concentration.

**Figure 14:**
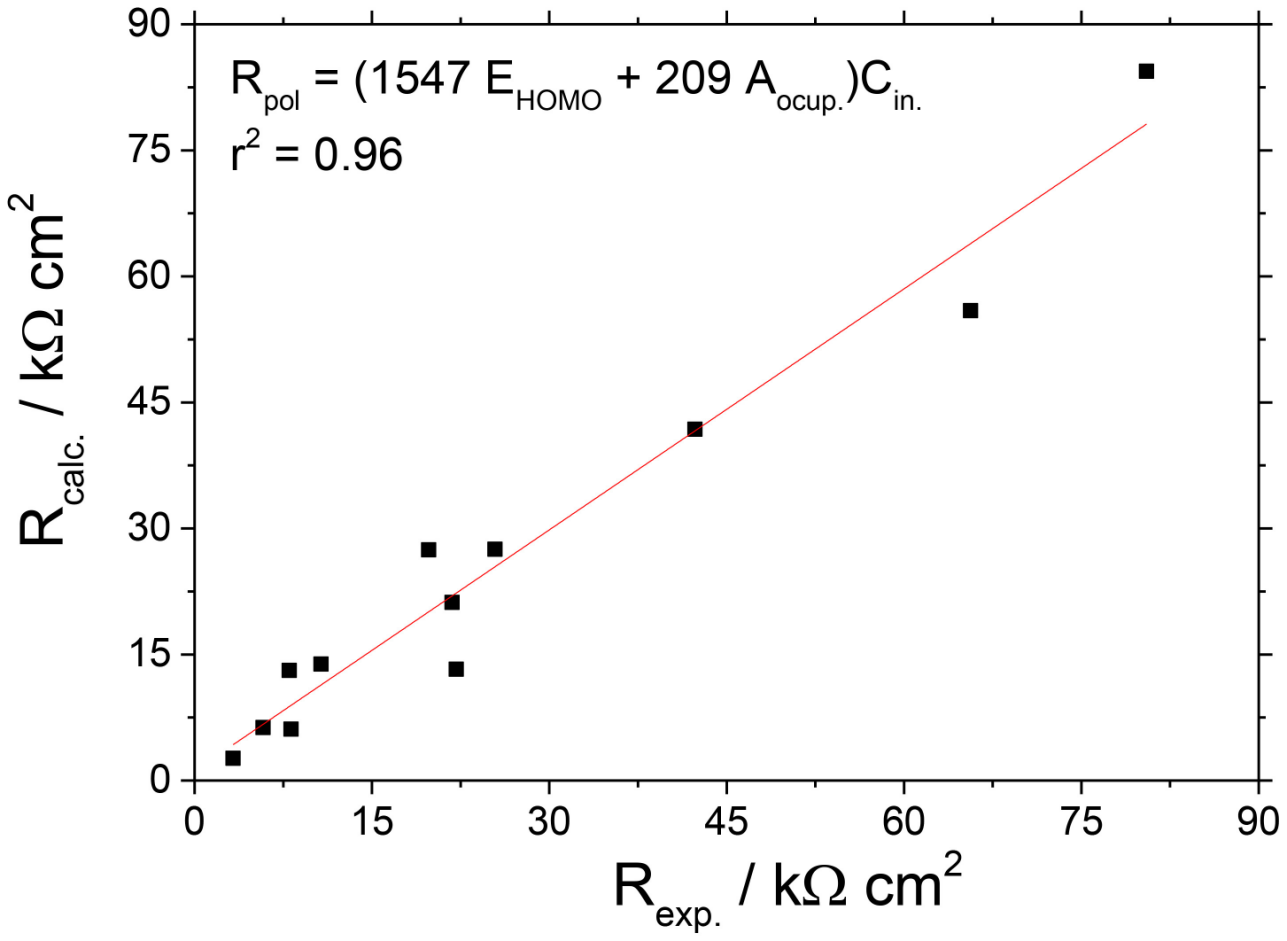
Correlation between the experimental and calculated *R*_p_ values for the caprylate salts, obtained by the linear multiple regression model.

Figure 14 shows the correlation of experimental and calculated values of *R*_p_ for the caprylate salts, which were obtained by linear regression model considering *E*_HOMO_ and the occupied area (A_ocup_). Figure 14 indicates that a *r*^2^ = 0.96 means that *E*_HOMO_ and size of the inhibitor (A_ocup_) can explain much of the adsorption process of caprylate salts.

The obtained equation shows that for caprylates more positive are *E*_HOMO_ and A_ocup_ higher is the polarization resistance of Zn. It means that the caprylates with higher ability to donate electrons associated with a relative great size, show higher tendency to be better corrosion inhibitors.

The correlation between experimental and theoretical data show that both data obtained from EIS measurements and theoretical calculations (DFT) agree about the efficiency of the inhibitor and also with the interaction energies of caprylate salts with the zinc surface. Therefore, theoretical methods are able to guide the choice of the best inhibitor prior to the experimental measurements. Both electrochemical impedance and DFT calculations showed that the larger the molecule the higher is the inhibition efficiency. Furthermore, it was observed that the EIS measurements and theoretical calculations agree with the humid chamber tests, in which the order of inhibition efficiency was: CDCHA > CCHA > CETA. However, only the EIS tests and theoretical calculations are not sufficient to indicate the VCI with higher performance because they depend on the parameters used in the calculation, the molecular weight and vapor pressure of inhibitor beyond the room temperature value.

## Conclusions

VCI were evaluated by EIS, quantum chemical calculations (DFT), humid chamber tests and surface characterization by SEM, EDS, and optical micrograph. These studies allowed to drawing the following conclusions:

- The quantum parameters (*E*_LUMO_ and *E*_HOMO_) indicated that the adsorption model involves electrons transfer from the VCI to the metal. The smallest difference between *E*_HOMO_ and *E*_LUMO_ for CDCHA and lowest interaction energy may explain its high efficiency of inhibition. Other factors like the lower solubility of the DCHA, the higher hydrophobic character, and steric effect due to the volume of the molecule and size factor also contributed to the performance of this VCI.
- The impedance spectra showed that the effectiveness of corrosion inhibition increased with augmentation of inhibitor concentration up to 10 × 10^−3^ or 15 × 10^−3^ mol L^−1^ and the CDCHA presented the greatest inhibition efficiency for zinc. The humid chamber tests corroborated the results about corrosion inhibition efficiency of CDCHA.
- The Dhar-Flory-Huggins' isotherm has better described the corrosion inhibitors performance due to the great influence of the size of the molecules to be adsorbed, and the $\Delta G_{\text{ads}}^{{}^{\circ}}$ values indicated chemical adsorption. Theoretical calculations may also help us to choose the better adsorption model.
- The quantitative structure and activity relationship (QSAR) of VCIs allowed associating the polarization resistance of each inhibitor with the frontier molecular orbital energy (HOMO) and the size of the inhibitor (A_ocup_).

## Author contributions

AB is the PhD who discussed ideas for theoretical studies, contributed to experimental design, and discussion of the results, and responsible for writing the manuscript. MV is the PhD student who has done the experimental measurements, data treatment, and helped in discussing, and write some parts of the manuscript. CF is the PhD who is specialist in volatile corrosion inhibitors, contributed to experimental design, and discussion of the experimental data. DT is the PhD who has executed the computational quantum calculations. DA is the PhD who has helped DT in discussing the correlation between experimental and theoretical results. GF is the PhD who planned and gave the theoretical support for calculations and discussion of theoretical studies, and helped to write the manuscript.

### Conflict of interest statement

The authors declare that the research was conducted in the absence of any commercial or financial relationships that could be construed as a potential conflict of interest.
