# Supplementary material for: Caprylate Salts Based on Amines as Volatile Corrosion Inhibitors for Metallic Zinc: Theoretical and Experimental Studies
Source: Front Chem. 2017 May 31;5:32. doi: 10.3389/fchem.2017.00032 (PMC5450003; doi:10.3389/fchem.2017.00032)
Supplement: Supplementary file 1 [file DataSheet1.DOCX]

***SUPPLEMENTARY INFORMATION***

**CAPRYLATE SALTS BASED ON AMINES AS VOLATILE CORROSION INHIBITORS FOR METALLIC ZINC: THEORETICAL AND EXPERIMENTAL STUDIES**

M. A. G. Valente Jr.^1^, D. A. Teixeira^1,2^, D. Lima Azevedo^3^, G. T. Feliciano^1^, A. V. Benedetti^1,*^, C. S. Fugivara^1^

*^1^Instituto de Química de Araraquara, Universidade Estadual Paulista “Júlio de Mesquita Filho” (UNESP), Araraquara-SP, Brazil.*

*^2^Instituto Federal do Mato Grosso, Campus Cuiabá (IFMT), Cuiabá, MT, Brazil.*

*^3^Instituto de Física, Universidade de Brasília (UNB), Brasília, DF, Brazil.*

*Corresponding author

E-mail address: benedeti@iq.unesp.br (A.V. Benedetti)

Energy dispersive X ray spectroscopy (EDS) analysis of deposits formed on zinc surfaces after 15 days immersed in 0.1 mol L^-1^ NaCl solution in (a) the presence of inhibitor (CDCHA) and (b) the absence of inhibitor.

(a)

**(b)**

**Figure S1.** The energy dispersive X ray microanalysis (EDS) of deposits formed on zinc surfaces after 15 days immersed in 0.1 mol L^-1^ NaCl solution: (a) in the presence of inhibitor and (b) in the absence.

Figure S2 shows Raman spectra for zinc surface after immersion in VCIs solution for 24 h.

(a)

(b)

(c)

**Figure S2.** Raman spectra for zinc surface after immersion in VCIs solution for 24 h: (a) Cyclehexylammonium caprylate; (b) dicyclehexylammonium caprylate; (c) ethanolammonium caprylate.

Table 1 shows the main bands obtained from the Raman spectra for zinc surface after 21 days of immersion in 0.1 mol l^-1^ NaCl containing VCI salts.

**Table S1.** Raman bands obtained for zinc surface after 21 days of immersion in 0.1 mol l^-1^ NaCl in the presence of ammonium caprylate salts: CCHA, CDCHA and CETA.

| **VCI** | **Wave number (υ) / cm^-1^** | **Attribution** | **reference** |
| --- | --- | --- | --- |
| CCHA | 2941, 2906, 2859, 1449, 1261, 1033, 848, 786, 551, 452 | CHA amine | a |
|  | 2670, 2859, 3227, 3326 | ZnO | d |
|  | 3480 | simonkoleite | e |
| CDCHA | 1180-1460 | DCHA amine/caprylate | b,c |
|  | 1123, 920 | caprylate | f |
|  | 1068 | Caprylate/ simonkoleite | c, e |
|  | 396 | ZnO |  |
| CETA | 1304, 1440 | caprylate | f |
|  | 381, 438, 1057 | ZnO | g, h, i, j, l. |
|  | 2704, 2856, 3220, 3324 | ZnO | d |
|  | 3480 | simonkoleite | e |

Isotherms of Dhar-Flory-Huggins, for amine derivatives caprylate salts used as volatile corrosion inhibitors of zinc and zinc oxides in 0.1 mol L^-1^ NaCl medium.

**Figure S3.** Isotherms of Dhar-Flory-Huggins for cyclohexylamine caprylate (CCHA), dicyclohexylamine caprylate (CDCHA) and ethanolamine caprylate (CETA).

**References**

a. CHEMICAL BOOK. Cyclohexylamine (108-91-8) Raman. 2008a. Available in:<http://www.chemicalbook.com/SpectrumEN_108-91-8_Raman.htm>. Accessed in: March, 6th, 2017.

b. Chemical Book. Dicyclehexylamine (101-83-7) Raman. 2008b. Available in:

<http://www.chemicalbook.com/SpectrumEN_101-83-7_Raman.htm>. Accessed in: March, 6th, 2017.

c. Chemical Book. Sodium octanoate (1984-06-1) Raman. 2008e. Available in:

<www.chemicalbook.com/SpectrumEN_1984-06-1_Raman.htm>. Accessed in: March, 6th, 2017.

d. Silva, C. F. P.; Duarte, M. L. T. S.; Fausto, R. (1999) A concerted SCF-MO ab initio and vibrational spectroscopic study of the conformational isomerism in 2-

aminoethanol. Journal of Molecular Structure, 482-483, 591-599.

e. Autengruber, R.; Luckeneder, G.; Hassel, A. W. (2012) Corrosion of press hardened galvanized steel. Corrosion Science, 63, 12-19.

f. Lin-Vien, D. et al. The handbook of infrared and Raman characteristic

frequencies of organic molecules. San Diego: Academic Press, 1991. 503 p.

g. Bernard, M. C.; Hugot-Le Goff, A.; Phillips, N. (1995) *In situ* Raman study of the corrosion of zinc-coated steel in the presence of chloride. I. Characterization and stability of zinc corrosion products. Journal of the Electrochemical Society,142, 2162-2167.

h. Cusco, R. et al. (2007. Temperature dependence of Raman scattering in ZnO. Physical Review B: Condensed Matter and Materials Physics, 75, 165202/1-165202/11.

i. Devaraj, R.; Karthikeyan, K.; Jeyasubramanian, K. (2013) Synthesis and

properties of ZnO nanorods by modified Pechini process. Applied Nanoscience,

3, 37-40.

j. Gandikota, V.; Xing, Y. (2014) Flame aerosol synthesis of freestanding ZnO nanorods. Advances in Nanoparticles, 3, 5-13.

l. Chemical Book. Ethanolamine (141-43-5) Raman. 2008c. Available in:

<http://www.chemicalbook.com/SpectrumEN_141-43-5_Raman.htm>. Accessed in: March, 6th, 2017.
